# Polymorphisms in Genes of Lipid Metabolism Are Associated with Type 2 Diabetes Mellitus and Periodontitis, as Comorbidities, and with the Subjects' Periodontal, Glycemic, and Lipid Profiles

**DOI:** 10.1155/2021/1049307

**Published:** 2021-11-11

**Authors:** Ingra G. Nicchio, Thamiris Cirelli, Rafael Nepomuceno, Marco A. R. Hidalgo, Carlos Rossa, Joni A. Cirelli, Silvana R. P. Orrico, Silvana P. Barros, Letícia H. Theodoro, Raquel M. Scarel-Caminaga

**Affiliations:** ^1^Department of Diagnosis and Surgery, São Paulo State University-UNESP, School of Dentistry at Araraquara, Araraquara, SP, Brazil; ^2^Department of Morphology, Genetics, Orthodontics and Pediatric Dentistry, São Paulo State University-UNESP, School of Dentistry at Araraquara, Araraquara, SP, Brazil; ^3^Advanced Research Center in Medicine, Union of the Colleges of the Great Lakes (UNILAGO), São José do Rio Preto, SP 15030-070, Brazil; ^4^Department of Periodontology, University of North Carolina at Chapel Hill-UNC, School of Dentistry, Chapel Hill, NC, USA; ^5^Department of Diagnosis and Surgery, São Paulo State University-UNESP, School of Dentistry at Araçatuba, Araçatuba, SP, Brazil

## Abstract

**Background:**

Type 2 diabetes mellitus (T2DM) and periodontitis (P) commonly occur as comorbidities, but the commonalities in the genetic makeup of affected individuals is largely unknown. Since dyslipidemia is a frequent condition in these individuals, we investigate the association of genomic variations in genes involved in lipid metabolism with periodontal, glycemic, lipid profiles, and the association with periodontitis and T2DM (as comorbidities).

**Methods:**

Based on clinical periodontal examination and biochemical evaluation, 893 subjects were divided into T2DM+P (T2DM subjects also affected by periodontitis, *n* = 205), periodontitis (*n* = 345), and healthy (*n* = 343). Fourteen single-nucleotide polymorphisms (SNPs) were investigated: *LDLR* gene (rs5925 and rs688), *APOB* (rs676210, rs1042031, and rs693), *ABCC8* (rs6544718 and 6544713), *LPL* (rs28524, rs3735964, and rs1370225), *HNF1A* (rs2650000), *APOE* (rs429358 and rs7412), and *HNF4A* (rs1800961). Multiple linear and logistic regressions (adjusted for covariates) were made for all populations and stratified by sex and smoking habits.

**Results:**

Individuals carrying *APOB*-rs1042031-CT (mainly women and never smokers) had a lower risk of developing periodontitis and T2DM (T2DM+P); altogether, this genotype was related with healthier glycemic, lipid, and periodontal parameters. Significant disease-phenotype associations with gene-sex interaction were also found for carriers of *APOB*-rs1676210-AG, *HNF4A*-rs1800961-CT, *ABCC8*-rs6544718-CT, *LPL*-rs13702-CC, and *LPL*-rs285-CT.

**Conclusions:**

Polymorphisms in lipid metabolism genes are associated with susceptibility to T2DM-periodontitis comorbidities, demonstrating gene-sex interaction. The *APOB*-rs1042031 was the most relevant gene marker related to glucose and lipid metabolism profiles, as well as with obesity and periodontitis.

## 1. Introduction

Periodontitis (P) is characterized by dysbiosis of the commensal microbiota associated with inflammation [1], which is responsible for the destruction of the hard and soft periodontal tissues that surround the tooth (connective gingival and alveolar bone tissue, cementum, periodontal ligament) causing dental mobility and culminating in tooth loss [2] It is considered the main cause of tooth loss in adults, affecting approximately 10-15% of the world population [3].

It derives from a complex and dynamic interplay among the immune system, microbiota, and lifestyle habits (e.g., smoking, and stress) ultimately resulting in homeostatic adaptation and maintenance of clinical health or disease manifestation. This interplay involves a highly complex network of host-derived molecules, which is largely determined by individual genomic variations. Individual genetic variations partly explain why manifestation of P often lacks correspondence with quality and amount of oral microbiota and also differs markedly between individuals with similar environmental backgrounds and comparable lifestyle habits [4].

Diabetes mellitus (DM) is a metabolic disorder that involves persistent hyperglycemia, resulting from defects in insulin production, insulin action, or both [5]. Type 2 diabetes mellitus (T2DM) occurs when there is a progressive loss of insulin secretion combined with insulin resistance [6, 7]. Overwhelming evidence indicates that T2DM is associated with increased prevalence and severity of P [8]. In fact, P is currently acknowledged as the sixth “classical” complication of T2DM [9].

Dyslipidemia is a deregulation of lipid metabolism, characterized by qualitative and/or quantitative changes in plasma lipoproteins and lipids [10]. Dyslipidemia is highly prevalent in T2DM [11], and T2DM is considered to cause dyslipidemia [12] and T2DM and dyslipidemia are closely related to cardiovascular complications, an important cause of morbidity and mortality [13]. A meta-analysis [14] supports the association between P and dyslipidemia, as the occurrence of P is significantly associated with reduced HDL levels and elevated serum concentrations of LDL and triglycerides. Thus, it is conceivable that dyslipidemia has a role mediating the interaction between T2DM and P, increasing the risks and severity of both conditions [15].

Periodontitis, T2DM, and dyslipidemia are complex conditions, which have in common their association with a systemic hyperinflammatory state [16]. In multifactorial diseases, genetic, epigenetic, and environmental factors play an essential role in their pathogenesis [17–19]. More studies are necessary to better understand the potential connection among P, T2DM, and dyslipidemia. Considering that the literature demonstrates a variety of genes relevant for dyslipidemia [20–28], we hypothesize that polymorphisms in genes of lipid metabolism could impact the occurrence of periodontitis alone or together with T2DM. Therefore, this study was aimed at investigating whether polymorphisms in lipid metabolism genes are associated with T2DM and periodontitis concomitantly present as comorbidities. Also, we investigate if specific genetic variants in these lipid metabolism genes are associated with glycemic and lipid profiles, as well with the periodontal status.

## 2. Materials and Methods

### 2.1. Study Subjects, Sample Collection, and DNA Isolation

A sample of 893 individuals with no familiar relations were included in the study from 1158 patients screened from the population who attended the Clinics of Periodontology, School of Dentistry at Araraquara, São Paulo State University (UNESP), Brazil, from 2010 to 2018. Subjects were excluded if they were younger than 30 years old, had chronic or systemic diseases (i.e., HIV infection or immunosuppressive chemotherapy), had received systemic antibiotics in the previous 3 months, were subjected to periodontal treatment in the previous 6 months, had less than 10 natural teeth (excluding third molars), and/or were pregnant or lactating.

The sample size was calculated with the G∗ Power Calculator, version 3.1.9 [29], as detailed in the Supplementary material. This study was approved by the Ethics in Human Research Committee of the School of Dentistry at Araraquara (UNESP; Protocol number 50/06) and followed the guidelines of the Helsinki Declaration of 1975 (revised in 1983).

The following biochemical parameters in the peripheral blood were determined for each participant: differential red and white blood cell count (ABX Micros 60); glycemic profile: insulin (chemiluminescence method), fasting glucose (modified Bondar and Mead method, Labtest Kit), and HbA1c (turbidimetric inhibition immunoassay method, Roche Kit); and lipid profiling: triglycerides (enzymatic-Trinder method, Labtest Kit), total cholesterol (enzymatic-Trinder method, Labtest Kit), HDL-cholesterol (high-density lipoprotein, enzymatic method, Labtest Kit), fractions of VLDL (very low-density lipoprotein), and LDL-cholesterol (low-density lipoprotein), calculated according to the Friedewald equation [7, 30, 31]. Moreover, the patients underwent a physical examination to assess waist circumference (cm), waist/hip ratio, body weight (kg), and height (m) to derive the body mass index (BMI) in kg/m^2^.

A complete periodontal examination of all subjects assessed the following clinical signs and parameters at four interproximal sites around each tooth: probing pocket depth (PPDi) and clinical attachment level (CALi), measured to the nearest millimeter by a UNC-15 periodontal probe (Millennium®) and the presence of visible plaque, marginal bleeding and bleeding on probing (BOP) [32, 33]. The last three parameters were registered as a percentage of the total number of interproximal sites assessed. All fully erupted teeth, except third molars and teeth with extensive destruction of the clinical crown, were examined. The criteria for the definition of periodontitis were according to the CDC/AAP (Centers for Disease Control and Prevention/American Academy of Periodontology) [4].

According to periodontal clinical examination and diagnosis of T2DM, the selected individuals were divided into three groups: T2DM+P (T2DM subjects also affected by periodontitis, *n* = 205), periodontitis (*n* = 345), and healthy (*n* = 343; no T2DM and no periodontitis). There were no individuals affected by only T2DM. Nondiabetics were considered as adequate when glycated hemoglobin (HbA1c) ≤ 5.6% and fasting glucose levels ≤ 99 mg/dL. Patients with periodontitis diagnosed by an endocrinologist as affected by T2DM were divided according to their glycemic metabolic control as poorly controlled (HbA1c ≥ 7.1%; *n* = 123) or well-controlled (HbA1c ≤ 7.0%; *n* = 82) [4].

A specific questionnaire confirmed smoking status. History of smoking was defined according to Miyake et al. as follows: (i) never smoked or (ii) ever smoked (including patients those who are current smokers and former smokers) [34].

Genomic DNA was extracted from buccal mucosa epithelial cells from each patient, using standard protocols involving proteinase K digestion, 8 M ammonium acetate, and subsequent ethanol precipitation [35]. The purity of genomic DNA was assessed on a microvolume UV spectrophotometer (NanoDrop® 2000, Thermo Scientific) and quantified on a fluorometer (Qubit® 2.0 fluorometer, Invitrogen). Samples were used whether they presented A_260/280_ ratio between 1.7 and 2.0, and the concentration was adjusted to 50 ng/*μ*L in TE buffer (Tris-HCl 10 mM, EDTA 0.1 M, SDS 0.5%—pH 8.0).

### 2.2. Polymerase Chain Reaction (PCR) Amplification and SNP Genotyping

The single-nucleotide polymorphisms (SNPs) in the *LDLR* (rs688, rs5925), *APOB* (rs676210, rs1042031, and rs676210), *ABCC8* (rs6544718 and rs6544713), *LPL* (rs285, rs3735964, and rs13702), *HNF1A* (rs2650000), *ApoE* (rs429358 and rs7412), and *HNF4A* (rs1800961) genes, selected for this study, were genotyped by Real-Time Polymerase Chain Reaction (PCR) using TaqMan® (Thermo Fischer Scientific, Foster City, CA, EUA) systems. The amplification was performed in a final volume of 12.5 *μ*L, containing 0.63 *μ*L of the specific TaqMan assay (Table 1) for genotyping each referred SNPs (including forward and reverse primers and probes marked with VIC and FAM), 6.25 *μ*L of TaqMan® Genotyping Master Mix (2X TaqMan®Genotyping Master Mix, Thermo Fisher Scientific), 20 ng of genomic DNA, and DNAse/RNAse free water in enough quantity to complete the final genotyping reaction. The following conditions were used for cycling the SNPs on a qPCR thermocycler (StepOnePlus Real-Time PCR System, Thermo Fisher Scientific): 95°C for 10 minutes, followed by 40 cycles of 95°C for 15 seconds, 60°C for 1 min. The genotyping analysis was performed with an online software (Applied Biosystems™ Analysis Software, Genotyping Analysis Module, version 3.2.).

### 2.3. Statistical Analysis

The general characteristics of each group were submitted to descriptive and analytical statistics using IBM SPSS Statistics 20.0 and GraphPad Prism 6.0. First, all data were evaluated by normality tests (D'Agostino-Pearson); then, they were applied with parametric (multiple comparisons with the one-way ANOVA followed by Tukey's test, expressed as the mean [±standard deviation]) or nonparametric (multiple comparisons by Kruskal-Wallis test followed by Dunn's test, expressed as the median minimum–maximum]) tests. Continuous demographic variables were compared among the groups using Student's *t*-test (for age). The chi-squared test (*X*^2^) was used for categorical variables (sex, ethnicity, and smoking).

The Hardy–Weinberg equilibrium (HWE) formula was applied to the genotype frequencies, separately for each group, using chi-square goodness-of-fit for any deviations from the expected genotype equilibrium. A chi-squared test (*X*^2^) was also used to assess differences in genotypic and allelic distributions of each SNP between the groups of patients. All these analyses and the minor allele frequency (MAF) of each SNP were calculated using the PLINK software [36].

Multiple logistic regression models assuming additive allelic effects, adjusting for age, sex and smoking habits (as independent variables) were used for testing associations between SNPs and the occurrence of only P (when comparing healthy and periodontitis groups), the occurrence of only T2DM (when comparing periodontitis and T2DM+P groups), and the occurrence of T2DM together with P (when comparing healthy and T2DM+P groups). These gene-phenotype associations were estimated by the odds ratio (OR) and confidence interval (CI) of 95%. The distributions of genotypes and alleles were compared in the two groups by chi-squared tests, and differences were considered significant when *p* < 0.05.

Furthermore, the same analysis was stratified by sex to identify the sex-specific effects of the SNPs on periodontitis by the additive model using multiple logistic regression adjusted by age and smoking. Similarly, to identify the effects of smoking habits with those SNPs on periodontitis, the population was stratified by smoking habits followed by the multiple logistic regressions adjusting by age and sex. Differences were considered significant when *p* < 0.05, but whether the Bonferroni correction is considered, the experiment-wise significance is the *p* value < 0.003, as determined by the total number of genotyped markers in the study (0.05/14 SNPs).

Spearman's correlations among glycemic, lipid, and periodontal parameters were made for each group (Supplementary Figure 1). A multiple linear regression was used to assess the relationship of each SNP independently with periodontal parameters (number of remaining teeth, marginal bleeding, BOPi, PPDi, and CALi) and with glycemic, lipid, and obesity parameters (fasting glucose, insulin level, HbA1c, triglycerides, total cholesterol, HDL, LDL, BMI, and waist-to-hip ratio). All these analyses and multiple logistic regressions were performed using the STATA software version 12.0 for Windows (Statistics/Data Analysis, Stata Corporation, College Station, TX, USA).

## 3. Results and Discussion

### 3.1. Results

Regarding the demographic characteristics, there were statistical differences among the groups in the mean of age, sex, and smoking habits. Healthy individuals were younger (mean 43 y.o.) (Table 2). Most of the investigated population was composed by female and never-smoker subjects. The T2DM+P group showed the highest percentage of visible plaque index, marginal bleeding, interproximal bleeding on probing (BOPi), interproximal probing pocket depth (PPDi ≥ 5 mm), and interproximal clinical attachment level (CALi 4-5 mm and CALi ≥ 6 mm), demonstrating worse clinical periodontal condition when compared to periodontitis (without diabetes) group. The highest fasting glucose and HbA1c levels in the T2DM+P group are compatible with the T2DM diagnosis criteria. Total cholesterol and triglycerides levels were higher in the T2DM+P group, as well as the body mass index (BMI) and waist-to-hip ratio (Table 2). Collectively, these results support an association or clustering of obesity/dyslipidemia/T2DM/periodontitis in this studied population.

In the Supplementary Table 1, besides the allelic and genotypic distributions among the groups, it is possible to note that the majority of SNPs presented a minor allele frequency (MAF) greater than 0.05. The genotype distributions of each SNP did not deviate from the Hardy–Weinberg equilibrium in the healthy group.

We performed multiple logistic regression analyses, using the additive model, to assess the association of each SNP with the pathological phenotype: periodontitis or T2DM together with P (T2DM+P) (Table 3). In the comparison between the healthy group versus Periodontitis, only the rs285 SNP (*LPL* gene) was significant, since heterozygous (CT) men had a 56% lower chance to develop periodontitis (OR = 0.44, 95% CI: 0.23–0.85, *p* = 0.01). Regarding the gene variation in the T2DM+P versus healthy, only the rs1042031 SNP in the *APOB* gene was statistically significant, as individuals with the CT genotype had a significantly lower chance to develop T2DM and periodontitis (adjusted OR 0.47, 95% CI: 0.28–0.77, *p* = 0.003) after Bonferroni correction. Female gender (adjusted OR 0.34, 95% CI: 0.18–0.65, *p* = 0.001) and never smoking subjects (adjusted OR 0.40, 95% CI: 0.21–0.75, *p* = 0.004) also were associated with reduced risk of T2DM+P. Even in the never-smoking female subjects, the *APOB*-rs1042031-CT genotype was still significantly associated with a lower chance of developing periodontitis and T2DM (adjusted OR 0.31, 95% CI: 0.15–0.69, *p* = 0.004). In the same *APOB* gene, never smokers carrying the rs676210-AG SNP had increased risk of T2DM+P (adjusted OR = 1.85, 95% CI: 1.06–3.20, *p* = 0.03). Regarding the *ABCC8* gene, men carrying the rs6544718-CT also showed less chance (63%) to develop T2DM+P (OR = 0.37; 95%CI = 0.16 − 0.83; *p* = 0.01). Homozygous (CC) men for the rare allele in the rs13702 (*LPL* gene) were more susceptible to T2DM+P (adjusted OR 4.38, 95% CI: 1.52–12.58, *p* = 0.006). Increased risk of periodontitis was also observed for all subjects carrying the rs180096-CT (*HNF4A* gene), since they were almost twice more susceptible to the development of T2DM+P (OR = 1.96; 95%CI = 1.01–3.80; *p* = 0.04) (Table 3).

Additional result analyses in the different groups are presented in the Supplementary material. Supplementary Figure 1 shows Spearman's correlations of biochemical and periodontal data in each studied group. In Supplementary Table 2, we present data of median (minimum–maximum) of glycemic and lipid profiles and physical exams of individuals belonging to the T2DM+P subgroups. In this Supplementary Table 2, when we subdivided T2DM+P by the severity of periodontitis, and we compared well-controlled T2DM+P with poorly controlled T2DM+P, we observed that the patients with severe periodontitis presented, in general, a higher glycemic and lipid biochemical levels. For example, poorly controlled with severe periodontitis T2DM+P patients showed the highest levels of fasting glucose and HbA1c.

When we considered T2DM_P moderate versus severe periodontitis patients compared by their lipid parameters, we observed in dyslipidemic T2DM patients with severe P statistically significant higher levels of total cholesterol, LDL-cholesterol, and triglycerides, suggesting that the severity of P could be related with the worst lipid profile of the patients.

Multiple linear regression analyses indicate that the *APOB-*rs1042031-CT was related to the number of remaining teeth (*β* = −0.08; *p* = 0.017), bleeding on probing (*β* = −0.05; *p* = 0.03), PPDi ≥ 5 mm (*β* = −0.08; *p* = 0.008), and CALi ≥ 6 mm (*β* = −0.07; *p* = 0.01) (Table 4). A negative coefficient suggests that as the independent variable increases, the dependent variable tends to decrease, thus supporting a possible protective role for this SNP in periodontitis. In contrast, the rs676210-AG SNP in the same *APOB* gene was positively associated with bleeding on probing (*β* = 0.05; *p* = 0.02) and CALi ≥ 6 mm (*β* = 0.07; *p* = 0.01). The SNP rs5925 (*LDLR* gene) was significantly related to marginal bleeding (*β* = −0.17; *p* = 0.02), which may suggest an influence on immune response to oral biofilm bacteria. Multiple linear regression analyses assessing the association of each SNP with biochemical and obesity parameters (Table 5) also detected the *APOB-*rs1042031-CT SNP as relevant for glycemic, lipid, and obesity parameters, particularly the waist-to-hip ratio (*β* = −0.25; *p* ≤ 0.01). The rs676210-AA SNP in the same *APOB* gene was positively related to fasting glucose (*β* = 0.21; *p* = 0.03), supporting a role for this genetic variation in T2DM. The rs285 SNP (*LPL* gene) was also related to BMI and to waist-to-hip ratio (Table 5). Results of Spearman's correlations between biochemical profiles and periodontal parameters for each group can be found in the Supplementary Figure 1.

### 3.2. Discussion

This case-control study demonstrates the association of polymorphisms in genes relevant for lipid metabolism with the occurrence of periodontitis (P) or the occurrence of T2DM and P concomitantly (T2DM+P). Specifically, the rs1042031 SNP in the *APOB* gene and the rs6544718 SNP in the *ABCC8* gene were protective, as their presence is associated with a significantly lower risk of T2DM+P. In contrast, the rs676210 (*APOB*), rs13702 (*LPL*), and rs1800961 (*HNF4A*) SNPs were associated with increased risk of T2DM+P. There was no significant association between SNPs in *LDLR* and *APOE* genes with either P or T2DM+P in Brazilian subjects.

Polymorphisms in the *APOB* gene have been associated with variability in serum cholesterol levels and coronary atherosclerosis in some populations [37, 38]. The rs1042031 SNP involves a silent mutation from cytosine to thymine in the third base of the 2488 codon. It has been associated with dyslipidemia [22, 39, 40], osteonecrosis [41], and ischemic stroke [23]. Here, we identified the association of *APOB*-rs1042031-CT with the lower chance of presenting P and T2DM concomitantly (T2DM+P). After the sample stratification by sex or smoking habits, we confirm these findings in females and in never-smoking individuals. Subsequent stratification by gender and smoking status (together) confirmed that *APOB*-rs1042031-CT females who never smoked had a lower chance to develop T2DM+P (*p* = 0.004). It was reported that the risk of having plaques on carotid arteries was lower in T2DM subjects carrying mutant specific alleles (E-alleles) [42]. The rs676210 SNP is a missense mutation involving G to A substitution that results in the conversion of proline to leucine, also named as Pro2739Leu. The rare allele A at the rs676210 SNP was significantly associated with the increased triglyceride-lowering effects of fenofibrate, a drug commonly used to treat hypertriglyceridemia [43], with reduced levels of LDL and triglycerides in Europeans [44]; and GWAS also associated this mutation with increased circulating oxidized LDL (oxLDL) [45]. We identified significant association of the *APOB*-rs676210-AG with presence of T2DM+P, whereas the same SNP in homozygosis (rs676210-AA) was associated with increased fasting glucose (OR = 0.21; 95%CI = 0.01 − 0.41; *p* = 0.03, Table 5) in our population.

Another relevant result found here was that the poorly controlled with severe periodontitis T2DM+P patients showed the highest levels of fasting glucose and HbA1c. It is worth to take into mind that 40% of the given plasma HbA1c pool reflects glycemia levels of the previous 31-90 days and 10% during the previous 91-120 days [46, 47] and that hyperglycemia inhibits new bone formation and exacerbates alveolar bone resorption due to periodontal disease [48].

Genetic variants in the *ABCC8* gene (OMIM: 600509) have also been widely explored in candidate genes case-control studies due to its function of encoding the sulfonylurea receptor, which plays an important role in insulin secretion [49]. Pharmacogenetics studies usually investigate this gene because of its relevance for the efficacy of hypoglycemic drugs, since the failure in oral drug therapy leads to poorly regulated glycemia and dyslipidemia [24, 50]. Thus, due to the relevance of *ABCC8* gene for glycemia in patients with T2DM, it is plausible that this gene may also affect the occurrence and severity of periodontitis in these patients, via poor metabolic control. From the two SNPs investigated in the *ABCC8* gene, rs6544718 showed a gender-biased positive association with a lower chance to develop T2DM+P, as men carrying rs6544718-CT were 63% less likely to develop T2DM+P. Another gender-biased genetic influence was observed for the *LPL* gene (OMIM 609708), with men carrying the rs13702-CC SNP presenting increased occurrence of T2DM+P. Interestingly, in a study enrolling patients affected by only T2DM, Hatefi et al. [25] reported the relationship between this CC genotype with a greater susceptibility to develop T2DM, in agreement with our results. The rs13702-CC may favor the entry of fatty acids into the liver, adipocytes, and skeletal muscle, which leads to the development of insulin resistance and, later, T2DM [25]. Another SNP in the same *LPL* gene, the rs285-CT, was associated with a lower chance of developing periodontitis. Moreover, the *LPL*-rs285-CC SNP was also related to the waist-to-hip ratio, as well as to BMI (Table 5).

Polymorphisms in the hepatocyte nuclear factor-4*α* (*HNF4A)* (OMIM: 60028) and HNF1 homeobox A (*HNF1A*) (OMIM 14210) genes were included in the present study because both genes encode important transcription factors involved in the regulation of lipid and glycemic metabolism [28]. *HNF4A* has been extensively associated with maturity-onset diabetes of the young (MODY) characterized by deficient pancreatic beta cell insulin secretion [51, 52], which was recently accurately revised by Banday et al. [53]. *HNF1A* is a transcription factor that interacts with *HNF4A* and leads to subtypes of MODY and, consequently, increases T2DM susceptibility. This gene resides in the T2DM-linked region on the chromosome 20 which supports a role as a positional candidate gene for the disease [51]. *HNF4A*-regulated proteins are involved in the pathogenesis of T2DM [54]. Patients with T2DM have been shown to have a characteristic deregulation of lipid metabolism, with lower levels of very low-density lipoprotein (VLDL) [26]. We identified that *HNF4A-*rs1800961-CT increases the risk of T2DM+P almost twofold. These patients are heterozygotes for the rare nonsynonymous mutation T130I (rs1800961). Other SNPs in the *HNF4A* gene were associated with elevated serum lipid levels and presence of metabolic syndrome, characterized by elevated glucose parameters in dyslipidemic Finnish and Mexican families [27].

There is no consensus regarding the sex-specific susceptibility to complex diseases in humans. Some studies had reported that men had the worst periodontal conditions [55, 56], and women with low carbohydrate intake were protected against development of T2DM [49]. Clinical [57], *in vitro* [58], and *in vivo* [59] studies have indicated the influence of steroid hormones on inflammation, immune response, and bone metabolism, demonstrating an association of periodontitis with sex hormone levels. More studies specifically designed to assess the sex or smoking habits are necessary to investigate the role of these SNPs in the context of the complex diseases here investigated.

The present case-control study was not age-sex matched, and the absence of a group with subjects affected by only T2DM were limitations of this study. Because of this fact, we are not able to associate a SNP with T2DM as an isolated disease. Since the population studied here was a convenience sample of patients seeking care/referred to the periodontics clinic, it was expected that these subjects were affected by periodontitis. Nevertheless, patients diagnosed by T2DM frequently have periodontitis, which may reflect the difficulty in achieving and sustaining proper metabolic control allowing for the influence of diabetes-related changes in angiogenesis, immune response, and repair mechanisms on the host-microbial interactions in the periodontal microenvironment. In fact, evidence supports that in well-controlled T2DM, the extent and severity of periodontitis, as well as the response to treatment, are similar to that of systemically healthy patients. Thus, it is possible that the duration of diabetes (i.e., time since diagnosis) and quality of metabolic control over time may have varied wildly in the studied subjects. Although we had mentioned the use of Bonferroni's correction, we did not strictly consider the 95% confidence for statistical significance. This was a compromise to reduce the chance of type II error [60–62], which would lead us to reject a true biological association hypothesis. Thus, our results should be interpreted with caution, since some of the *p* values lower than 0.05 may in fact reflect type I errors (false positives).

Although the sample size allowed for adequate statistical power, it is important to expand the findings on these genetic variants in larger Brazilian populations from different regions of the country and in multiple ethnic populations throughout the world, in agreement with Laine et al. [63]

## 4. Conclusions

In conclusion, polymorphisms in genes involved in lipid metabolism are associated with susceptibility to the occurrence of T2DM-Periodontitis, with a gene-sex interaction. The *APOB*-rs1042031 was the most relevant gene marker related to glucose and lipid metabolism profiles, as well as with clinical obesity and periodontitis. Further studies are necessary to confirm these results, investigating the potential functional role of those SNPs at transcriptional and translational levels, and the potential influence of sex in this context.

## Figures and Tables

**Table 1:** Information regarding the 14 investigated single-nucleotide polymorphisms (SNPs) in lipid metabolism genes.

SNP	Assay ID	SNP position^†^, alleles	Gene and classification of mutation	Call rate (%)^∗^
rs5925	C___2804279_10	chr19:g.11230881T>C	*LDLR* (silent mutation)	96.92
rs688	C___2804264_20	chr19:g.11227602C>T	*LDLR* (silent mutation)	96.36
rs676210	C___3216558_10	chr11:g.21231524G>A	*APOB* (missense mutation)	95.66
rs1042031	C___7615381_20	chr11:g.21225753C>T	*APOB* (silent mutation)	96.22
rs693	C___7615420_20	chr11:g.21232195G>A	*APOB* (silent mutation)	96.92
rs6544718	C__25642779_10	chr2:g.44104925T>A	*ABCC8* (missense variant)	97.21
rs6544713	C__26135636_10	chr2:g.44073881T>C	*ABCC8* (intron variant)	97.35
rs285	C__12104266_10	chr8:g.19815189C>T	*LPL* (intron variant)	96.65
rs3735964	C__25800209_10	chr8:g.19824045C>A	*LPL* (3 prime UTR variant)	96.51
rs13702	C___9639448_10	chr8:g.19824492T>A	*LPL* (3 prime UTR variant)	90.08
rs2650000	C__15874272_10	chr12:g.121388962A>C	*HNF1A* (intragenic)	96.51
rs429358	C___3084793_20	chr19:g.45411941T>C	*APOE* (missense variant)	97.35
rs7412	C____904973_10	chr19:g.45412079C>T	*APOE* (missense variant)	97.35
rs1800961	C___7591528_10	chr20:g.43042364C>T	*HNF4A* (missense variant)	96.51

^†^Genome Reference Consortium Human Build 37 (GRCh37.p13) or hg19. ^∗^Represents the percentage of genotyping performed with success (all subjects).

**Table 2:** Demographic characteristics of the studied groups, clinical periodontal parameters, glycemic and lipid profiles, and physical exams of all groups.

	Healthy	Periodontitis	T2DM+P
Demographic characteristics	*n* = 343	*n* = 345	*n* = 205
*Age, mean (±SD)*	43 (±10.88)^a^	49.06 (±9.29)^b^	56.03 (±9.49)^c^
*Sex,n(%)*			
Male	110 (32%)^a^	204 (59%)^b^	86 (42%)^c^
Female	233 (68%)^a^	141 (41%)^b^	119 (58%)^c^
*Smoking habits,n(%)*			
Smoker	29 (8.45%)^a^	34 (9.85)^a^	20 (10%)^a^
Former smoker	47 (13.7%)^a^	89 (25.8%)^a^	64 (31%)^a^
Never smoker	267 (77.85%)^a^	222 (64.35%)^b^	121 (59%)^b^
*Periodontal parameters, mean (±SD)*			
Number of teeth	25.5(±3.43)^a^	22.84 (±4.48)^b^	20.54 (±5.51)^c^
Visible plaque index (% site)	24.75(±20.0)^a^	54.82 (±28.13)^b^	56.53(±26.96)^c^
Marginal bleeding (% site)	8.79(±11.72)^a^	20.07(±23.25)^b^	40.63 (±23.91)^c^
BOPi (% site)	3.30 (±4.98)^a^	39.98 (±23.17)^b^	51.03 (±27.16)^c^
PPDi ≤ 4 mm (% site)	99.93 (±0.30)^a^	86.87 (±14)^b^	79.11 (±22.69)^c^
PPDi ≥ 5 mm (% site)	0.07 (±0.30)^a^	13.13 (±14.0)^b^	20.89 (±22.89)^c^
CALi ≤ 3 mm (% site)	99.49 (±0.78)^a^	68.40 (±21.39)^b^	56.22 (±27.32)^c^
CALi = 4–5 mm (% site)	0.46 (±0.75)^a^	20.07 (±11.92)^b^	24.99 (±15.23)^c^
CALi ≥ 6 mm (% site)	0.04 (±0.23)^a^	11.53 (±13.68)^b^	18.79 (±20.79)^c^
*Biochemical and physical data median (minimum–maximum)*			
Fasting blood glucose (mg/dL)	91.50 (80.0-99)^a^	92 (60.0-99)^a^	152.2 (83.0-467.9)^b^
HbA_1c_ (%)	5.4 (4.8-5.6)^a^	5.6 (4.4-5.60)^a^	7.9 (4.5-14.30)^b^
Insulin (UI/mL)	7.3 (3.6-36.5)^a^	9.8 (4.1-31.3)^a^	13.5 (3.2-159.2)^b^
Total cholesterol (mg/dL)	176 (134-218)^a^	176 (137-223)^a^	192.5 (139-306)^b^
HDL-cholesterol (mg/dL)	56 (42-84)^a^	53 (30-88)^a^	44 (24-350.6)^b^
LDL-cholesterol (mg/dL)	96.40 (50.6-155.8)^a^	96.20 (17.0-732.0)^a^	109.8 (7.9-525.0)^a^
Triglycerides (mg/dL)	104.4 (41-283.8)^a^	133 (47.5-709.4)^b^	156.2 (33.9-765.0)^b^
BMI (kg/m^2^)	27.81 (18.9-41.6)^a^	26.9 (18.21-37.9)^a^	29.82(18.5-49.9)^b^
Waist-to-hip ratio (cm)	0.88 (0.09-1.09)^a^	0.91 (0.74-1.07)^a^	0.95 (0.76-1.25)^b^

SD: standard deviation; BOPi: interproximal bleeding on probing; P: periodontitis; PPDi: interproximal probing pocket depth; CALi: interproximal clinical attachment level. Chi-squared test was used to determine differences among groups for sex and smoking habits. ^a,b,c^Different letters means *p* value < 0.05; periodontal parameters were compared by one-way ANOVA, followed by Tukey's test. Biochemical and lipid levels were compared by Kruskal-Wallis test, followed by Dunn's test.

**(a):** 

Gene	Healthy versus periodontitis		Healthy versus T2DM+P		Periodontitis versus T2DM+P	
*SNP*						
LDLR	Adj OR (95% CI)	*p* value	Adj OR (95% CI)	*p* value	Adj OR (95% CI)	*p* value
*rs5925*						
All patients^†^	Adjusted OR^†^		Adjusted OR^†^		Adjusted OR^†^	
TT	Ref		Ref		Ref	
CT	0.96 (0.68–1.36)	0.81	1.01 (0.62–1.62)	0.97	1.13 (0.73–1.75)	0.58
CC	0.92 (0.58–1.46)	0.72	1.12 (0.60–2.06)	0.71	1.02 (0.58–1.79)	0.93
*X* ^2^						
Genotype	0.096		0.355		0.183	
Allele	0.031		**0.003**		**0.041**	
Male^‡^	Adjusted OR^‡^		Adjusted OR^‡^		Adjusted OR^‡^	
TT	Ref		Ref		Ref	
CT	1.31 (0.73–2.37)	0.36	1.21 (0.50–2.89)	0.67	1.15 (0.56–2.36)	0.70
CC	1.13 (0.51–2.50)	0.76	2.50 (0.80–7.82)	0.11	1.07 (0.45–2.60)	0.86
Female^‡^						
TT	Ref		Ref		Ref	
CT	0.82 (0.53–1.27)	0.38	0.95 (0.53–1.69)	0.85	1.21 (0.64–1.94)	0.68
CC	0.83 (0.47–1.47)	0.52	0.81 (0.38–1.72)	0.59	0.99 (0.44–2.05)	0.97
Never smoking^¶^	Adjusted OR^¶^		Adjusted OR^¶^		Adjusted OR^¶^	
TT	Ref		Ref		Ref	
CT	0.91 (0.61–1.38)	0.68	1.04 (0.59–1.86)	0.87	1.16 (0.66–2.01)	0.59
CC	0.97 (0.57–1.70)	0.96	1.20 (0.57–2.50)	0.61	1.11 (0.55–2.23)	0.31
Ever smoking^¶^						
TT	Ref		Ref		Ref	
CT	1.07 (0.55–2.09)	0.83	0.86 (0.36–2.08)	0.73	1.01 (0.48–2.11)	0.97
CC	0.79 (0.32–1.89)	0.59	0.93 (0.30–2.87)	0.91	0.86 (0.33–2.22)	0.18
*rs688*						
All patients^†^	Adjusted OR^†^		Adjusted OR^†^		Adjusted OR^†^	
CC	Ref		Ref		Ref	
CT	0.91 (0.65–1.29)	0.60	0.83 (0.52–1.35)	0.47	0.96 (0.62–1.49)	0.87
TT	0.88 (0.55–1.42)	0.61	0.97 (0.52–1.82)	0.93	0.90 (0.50–1.62)	0.74
*X* ^2^						
Genotype	0.141		0.662		0.280	
Allele	0.070		0.116		**0.014**	
Male^‡^	Adjusted OR^‡^		Adjusted OR^‡^		Adjusted OR^‡^	
CC	Ref		Ref		Ref	
CT	1.30 (0.73–2.33)	0.37	1.15 (0.49–2.65)	0.75	0.99 (0.50–1.98)	0.99
TT	0.99 (0.45–2.21)	0.99	1.47 (0.47–4.56)	0.50	0.81 (0.32–2.05)	0.67
Female^‡^						
CC	Ref		Ref		Ref	
CT	0.76 (0.49–1.16)	0.48	0.73 (0.40–1.32)	0.32	0.94 (0.54–1.65)	0.84
TT	0.83 (0.46–1.50)	0.55	0.83 (0.39–1.80)	0.65	0.98 (0.46–2.08)	0.96
Never smoking^¶^	Adjusted OR^¶^		Adjusted OR^¶^		Adjusted OR^¶^	
CC	Ref		Ref		Ref	
CT	0.90 (0.60–1.36)	0.63	0.84 (0.47–1.50)	0.55	0.91 (0.52–1.58)	0.73
TT	0.95 (0.54–1.65)	0.54	1.06 (0.50–2.32)	0.87	0.99 (0.46–2.04)	0.99
Ever smoking^¶^						
CC	Ref		Ref		Ref	
CT	0.92 (0.48–1.77)	0.80	0.76 (0.32–1.81)	0.53	0.95 (0.46–1.97)	0.90
TT	0.73 (0.29–1.81)	0.50	0.78 (0.24–2.51)	0.67	0.73 (0.27–1.99)	0.54

**(b):** 

*APOB*	Adj OR (95% CI)	*p* value	Adj OR (95% CI)	*p* value	Adj OR (95% CI)	*p* value
rs676210						
All patients^†^	Adjusted OR^†^		Adjusted OR†		Adjusted OR^†^	
GG	Ref		Ref		Ref	
AG	1.30 (0.91–1.84)	0.15	1.49 (0.93–2.37)	0.09	1.10 (0.72–1.67)	0.65
AA	1.10 (0.38–2.19)	0.78	0.43 (0.13–1.41)	0.16	0.43 (0.16–1.15)	0.09
*X* ^2^						
Genotype	1.867		2.435		1.873	
Allele	1.926		0.262		0.438	
Male^‡^	Adjusted OR^‡^		Adjusted OR^‡^		Adjusted OR^‡^	
GG	Ref		Ref		Ref	
AG	1.10 (0.61–2.01)	0.74	1.23 (0.54–2.79)	0.62	1.26 (0.64–2.46)	0.49
AA	1.59 (0.58–4.38)	0.36	0.59 (0.10–3.44)	0.56	0.54 (0.15–1.90)	0.33
Female^‡^						
GG	Ref		Ref		Ref	
AG	1.43 (0.92–2.21)	0.11	0.63 (0.92–2.87)	0.09	0.99 (0.58–1.69)	0.99
AA	0.75 (0.28–2.02)	0.57	0.28 (0.05–1.66)	0.16	0.30 (0.05–1.63)	0.16
Never smoking^¶^	Adjusted OR^¶^		Adjusted OR^¶^		Adjusted OR^¶^	
	Ref		Ref		Ref	
AG	1.36 (0.90–2.04)	0.14	**1.85 (1.06–3.20)**	**0.03**	1.13 (0.68–1.89)	0.63
AA	0.81 (0.36–1.83)	0.61	0.46 (0.11–1.86)	0.27	0.59 (0.15–2.32)	0.45
Ever smoking^¶^						
GG	Ref		Ref		Ref	
AG	1.15 (0.58–2.28)	0.68	0.80 (0.33–1.94)	0.62	1.03 (0.49–2.15)	0.94
AA	2.93 (0.59–14.49)	0.19	0.23 (0.02–2.58)	0.23	**0.20 (0.04–0.96)**	**0.04**
*rs1042031*						
All patients^†^	Adjusted OR^†^		Adjusted OR^†^		Adjusted OR^†^	
CC	Ref		Ref		Ref	
CT	0.73 (0.52–1.03)	0.06	**0.47 (0.28–0.77)**	**0.003** ^∗^	0.75 (0.47–1.18)	0.21
TT	0.65 (0.23–1.85)	0.42	1.38 (0.46–4.12)	0.57	2.40 (0.79–7.21)	0.12
*X* ^2^						
Genotype	3.187		9.236		4.663	
Allele	2.875		3.167		0.158	
Male^‡^	Adjusted OR^‡^		Adjusted OR^‡^		Adjusted OR^‡^	
CC	Ref		Ref		Ref	
CT	0.92 (0.51–1.64)	0.78	0.78 (0.33–1.82)	0.56	0.96 (0.47–1.92)	0.90
TT	0.34 (0.06–2.08)	0.24	0.92 (0.12–6.72)	0.93	2.56 (0.32–19.92)	0.36
Female^‡^						
CC	Ref		Ref		Ref	
CT	0.64 (0.92–2.21)	0.11	**0.34 (0.18–0.65)**	**0.001** ^∗^	0.62 (0.33–1.13)	0.12
TT	0.92 (0.25–3.33)	0.89	1.74 (0.45–6.60)	0.41	2.29 (0.64–8.30)	0.20
Never smoking ^¶^	Adjusted OR^¶^		Adjusted OR^¶^		Adjusted OR^¶^	
CC	Ref		Ref		Ref	
CT	0.70 (0.47–1.06)	0.09	**0.40 (0.21–0.75)**	**0.004**	0.77 (0.42–1.40)	0.39
TT	0.55 (0.15–2.01)	0.37	1.19 (0.29–4.81)	0.80	1.96 (0.45–8.60)	0.37
Ever smoking^¶^						
CC	Ref		Ref		Ref	
CT	0.78 (0.41–1.46)	0.43	0.80 (0.41–1.46)	0.43	0.64 (0.30–1.34)	0.64
TT	0.89 (0.14–5.64)	0.90	0.89 (0.14–5.64)	0.90	3.84 (0.69–21.39)	0.12
Female+never smoking^¥^						
CC	Ref		Ref		Ref	
CT	1.13 (0.45–2.82)	0.78	**0.31 (0.15–0.69)**	**0.004**	0.55 (0.25–1.17)	0.12
TT	0.57 (0.03–9.61)	0.69	1.93 (0.43–8.60)	0.38	2.56 (0.52–12.49)	0.24
*rs693*						
All patients^†^	Adjusted OR^†^		Adjusted OR^†^		Adjusted OR^†^	
GG	Ref		Ref		Ref	
AG	1.05 (0.74–1.48)	0.77	0.98 (0.62–1.55)	0.93	0.95 (0.62–1.43)	0.80
AA	0.83 (0.52–1.33)	0.44	0.90 (0.48–1.69)	0.75	0.82 (0.45–1.48)	0.52
*X* ^2^						
Genotype	0.568		0.955		0.137	
Allele	**0.032**		0.154		0.057	
Male^‡^	Adjusted OR^‡^		Adjusted OR^‡^		Adjusted OR^‡^	
GG	Ref		Ref		Ref	
AG	0.91 (0.51–1.64)	0.76	0.67 (0.29–1.51)	0.33	0.75 (0.38–1.48)	0.41
AA	1.02 (0.49–2.15)	0.94	0.79 (0.27–2.29)	0.67	0.51 (0.21–1.24)	0.14
Female^‡^						
GG	Ref		Ref		Ref	
AG	1.43 (0.92–2.21)	0.11	1.19 (0.68–2.09)	0.52	1.10 (0.64–1.88)	0.71
AA	0.76 (0.28–2.02)	0.57	0.95 (0.43–2.11)	0.91	1.23 (0.55–2.74)	0.60
Never smoking^¶^	Adjusted OR^¶^		Adjusted OR^¶^		Adjusted OR^¶^	
GG	Ref		Ref		Ref	
AG	1.22 (0.81–1.83)	0.33	1.07 (0.62–1.86)	0.25	1.04 (0.61–1.75)	0.88
AA	0.77 (0.44–1.36)	0.37	0.91 (0.43–1.96)	0.82	1.01 (0.48–2.14)	0.97
Ever smoking^¶^						
GG	Ref		Ref		Ref	
AG	0.72 (0.37–1.39)	0.33	0.85 (0.36–1.96)	0.70	0.72 (0.35–1.47)	0.37
AA	0.91 (0.38–2.21)	0.85	0.91 (0.29–2.83)	0.87	0.63 (0.24–1.63)	0.34

**(c):** 

*ABCC8*	Adj OR (95% CI)	*p* value	Adj OR (95% CI)	*p* value	Adj OR (95% CI)	*p* value
*rs6544718*						
All patients^†^	Adjusted OR^†^		Adjusted OR^†^		Adjusted OR^†^	
CC	Ref		Ref		Ref	
CT	0.87 (0.60–1.25)	0.45	0.71 (0.45–1.12)	0.15	0.93 (0.57–1.52)	0.79
TT	1.21 (0.43–3.39)	0.71	0.64 (0.15–2.70)	0.54	0.42 (0.10–1.73)	0.23
*X* ^2^						
Genotype	1.023		NA		NA	
Allele	0.072		0.901		1.377	
Male^‡^	Adjusted OR^‡^		Adjusted OR^‡^		Adjusted OR^‡^	
CC	Ref		Ref		Ref	
CT	0.75 (0.41–1.37)	0.35	**0.37 (0.16–0.83)**	**0.01**	0.74 (0.29–1.87)	0.52
TT	0.75 (0.10–5.45)	0.77	0.89 (0.12–6.19)	0.90	1.96 (0.16–22.84)	0.59
Female^‡^						
CC	Ref		Ref		Ref	
CT	0.95 (0.60–1.50)	0.84	1.01 (0.57–1.77)	0.96	1.06 (0.60–1.89)	0.82
TT	1.43 (0.42–4.76)	0.55	0.40 (0.04–3.99)	0.44	0.17 (0.02–1.55)	0.11
Never smoking^¶^	Adjusted OR^¶^		Adjusted OR^¶^		Adjusted OR^¶^	
CC	Ref		Ref		Ref	
CT	0.90 (0.59–1.38)	0.65	0.69 (0.38–1.27)	0.24	0.66 (0.36–1.20)	0.17
TT	0.59 (0.14–2.39)	0.46	1.36 (0.08–22.70)	0.83	0.15 (0.01–1.35)	0.09
Ever smoking^†¶^						
CC	Ref		Ref		Ref	
CT	0.78 (0.39–1.54)	0.47	0.72 (0.36–1.44)	0.35	1.88 (0.71–4.98)	0.20
TT	3.94 (0.46–33.82)	0.21	0.51 (0.89–2.91)	0.45	2.46 (0.29–20.84)	0.40
*rs6544713*						
All patients^†^	Adjusted OR^†^		Adjusted OR^†^		Adjusted OR^†^	
CC	Ref		Ref		Ref	
CT	1.06 (0.76–1.49)	0.69	0.93 (0.61–1.41)	0.74	1.04 (0.66–1.62)	0.86
TT	1.10 (0.61–1.98)	0.74	1.30 (0.63–2.67)	0.46	1.21 (0.59–2.4)	0.59
*X* ^2^						
Genotype	0.379		0.491		0.081	
Allele	0.384		0.504		**0.032**	
Male^‡^	Adjusted OR^‡^		Adjusted OR^‡^		Adjusted OR^‡^	
CC	Ref		Ref		Ref	
CT	1.04 (0.59–1.81)	0.84	0.75 (0.39–1.45)	0.40	0.73 (0.34–1.56)	0.42
TT	0.67 (0.22–2.03)	0.48	0.61 (0.16–2.30)	0.47	1.32 (0.29–6.06)	0.71
Female^‡^						
CC	Ref		Ref		Ref	
CT	1.06 (0.69–1.62)	0.78	1.07 (0.62–1.83)	0.80	1.25 (0.71–2.18)	0.42
TT	1.34 (0.66–2.71)	0.40	1.90 (0.79–4.57)	0.15	1.23 (0.55–2.74)	0.59
Never smoking^¶^	Adjusted OR^¶^		Adjusted OR^¶^		Adjusted OR^¶^	
CC	Ref		Ref		Ref	
CT	0.89 (0.60–1.33)	0.58	0.88 (0.51–1.50)	0.64	0.84 (0.49–1.44)	0.54
TT	1.02 (0.51–2.02)	0.94	1.46 (0.57–3.71)	0.41	0.86 (0.36–2.04)	0.73
Ever smoking^†¶^						
CC	Ref		Ref		Ref	
CT	1.70 (0.88–3.26)	0.10	1.00 (0.51–1.94)	0.99	1.52 (0.66–3.53)	0.32
TT	1.29 (0.39–4.20)	0.67	1.06 (0.34–3.27)	0.91	2.84 (0.73–10.95)	0.12

**(d):** 

*LPL*	Adj OR (95% CI)	*p* value	Adj OR (95% CI)	*p* value	Adj OR (95% CI)	*p* value
*rs285*						
All patients^†^	Adjusted OR†		Adjusted OR^†^		Adjusted OR^†^	
TT	Ref		Ref		Ref	
CT	0.87 (0.60–1.26)	0.48	0.71 (0.44–1.14)	0.16	0.88 (0.53–1.43)	0.61
CC	0.71 (0.46–1.11)	0.13	0.86 (0.50–1.46)	0.58	0.95 (0.55–1.66)	0.88
*X* ^2^						
Genotype	1.566		1.76		1.166	
Allele	1.445		0.144		0.399	
Male^‡^	Adjusted OR^‡^		Adjusted OR^‡^		Adjusted OR^‡^	
TT	Ref		Ref		Ref	
CT	**0.44 (0.23–0.85)**	**0.01**	0.62 (0.29–1.33)	0.22	0.91 (0.39–2.11)	0.83
CC	0.64 (0.29–1.37)	0.25	0.49 (0.20–1.22)	0.13	0.71 (0.26–1.93)	0.51
Female^‡^						
TT	Ref		Ref		Ref	
CT	1.22 (0.76–1.94)	0.39	0.76 (0.41–1.39)	0.38	0.83 (0.45–1.54)	0.45
CC	0.74 (0.43–1.29)	0.29	1.17 (0.60–2.29)	0.62	1.06 (0.54–2.08)	0.85
Never smoking^¶^	Adjusted OR^¶^		Adjusted OR^¶^		Adjusted OR^¶^	
TT	Ref		Ref		Ref	
CT	0.94 (0.60–1.46)	0.78	0.79 (0.38–1.30)	0.26	0.95 (0.52–1.74)	0.88
CC	0.90 (0.53–1.50)	0.69	0.93 (0.48–1.79)	0.83	1.16 (0.60–2.21)	0.64
Ever smoking^†¶^						
TT	Ref		Ref		Ref	
CT	0.70 (0.34–1.44)	0.34	0.69 (0.33–1.47)	0.34	0.53 (0.20–1.39)	0.20
CC	**0.38 (0.16–0.89)**	**0.02**	0.75 (0.29–1.93)	0.55	1.04 (0.18–1.78)	0.33
*rs3735964*						
All patients^†^	Adjusted OR^†^		Adjusted OR^†^		Adjusted OR^†^	
CC	Ref		Ref		Ref	
AC	0.76 (0.50–1.14)	0.19	1.12 (0.67–1.86)	0.64	1.10 (0.62–1.87)	0.70
AA	0.77 (0.21–2.82)	0.69	1	—	1	—
*X* ^2^						
Genotype	NA		NA		NA	
Allele	1.738		0.767		**0.045**	
Male^‡^	Adjusted OR^‡^		Adjusted OR^‡^		Adjusted OR^‡^	
CC	Ref		Ref		Ref	
AC	0.62 (0.31–1.24)	0.18	1.95 (0.88–2.40)	0.09	1.15 (0.49–2.68)	0.74
AA	1	—	—	—	1	—
Female^‡^						
CC	Ref		Ref		Ref	
AC	0.83 (0.50–1.39)	0.48	1.07 (0.62–1.83)	0.80	1.02 (0.51–2.06)	0.93
AA	0.62 (0.15–2.57)	0.51	1.90 (0.79–4.57)	0.15	1	—
Never smoking^¶^	Adjusted OR^¶^		Adjusted OR^¶^		Adjusted OR^¶^	
CC	Ref		Ref		Ref	
AC	0.69 (0.42–1.14)	0.15	1.77 (0.94–3.33)	0.07	1.29 (0.71–2.35)	0.39
AA	0.60 (0.14–2.48)	0.48	1	—	1	—
Ever smoking^†¶^						
CC	Ref		Ref		Ref	
AC	0.93 (0.44–1.96)	0.84	0.52 (0.21–1.27)	0.15	0.60 (0.19–1.89)	0.38
AA	1	**—**	—	—	1	—
*rs13702*						
All patients^†^	Adjusted OR^†^		Adjusted OR^†^		Adjusted OR^†^	
TT	Ref		Ref		Ref	
CT	0.88 (0.62–1.25)	0.49	0.97 (0.62–1.52)	0.98	0.74 (0.46–1.20)	0.23
CC	1.07 (0.62–1.83)	0.80	1.20 (0.62–2.34)	0.57	1.44 (0.70–2.98)	0.31
*X* ^2^						
Genotype	0.695		2.215		1.092	
Allele	0.581		0.547		**0.011**	
Male^‡^	Adjusted OR^‡^		Adjusted OR^‡^		Adjusted OR^‡^	
TT	Ref		Ref		Ref	
CT	0.77 (0.42–1.39)	0.39	1.92 (0.92–3.99)	0.08	0.86 (0.36–2.04)	0.73
CC	1.26 (0.51–3.11)	0.61	**4.38 (1.52–12.58)**	**0.006**	2.17 (0.69–6.81)	0.18
Female^‡^						
TT	Ref		Ref		Ref	
CT	0.94 (0.60–1.46)	0.79	0.64 (0.36–1.13)	0.68	0.68 (0.38–1.20)	0.18
CC	0.98 (0.50–1.93)	0.96	0.47 (0.18–1.20)	0.11	1.10 (0.40–2.97)	0.85
Never smoking^¶^	Adjusted OR^¶^		Adjusted OR^¶^		Adjusted OR^¶^	
TT	Ref		Ref		Ref	
CT	0.74 (0.49–1.12)	0.16	0.98 (0.56–1.73)	0.95	0.81 (0.46–1.42)	0.47
CC	0.79 (0.43–1.46)	0.46	0.74 (0.30–1.85)	0.53	0.92 (0.37–2.26)	0.86
Ever smoking^†¶^						
TT	Ref		Ref		Ref	
CT	1.35 (0.70–2.59)	0.36	1.02 (0.49–2.11)	0.94	0.56 (0.23–1.39)	0.21

**(e):** 

*HNF1A*	Adj OR (95% CI)	*p* value	Adj OR (95% CI)	*p* value	Adj OR (95% CI)	*p* value
*rs2650000*						
All patients^†^	Adjusted OR^†^		Adjusted OR^†^		Adjusted OR^†^	
CC	Ref		Ref		Ref	
AC	0.82 (0.59–1.15)	0.26	1.18 (0.78–1.79)	0.42	0.80 (0.51–1.24)	0.32
AA	0.87 (0.49–1.52)	0.63	0.96 (0.49–1.85)	0.90	1.14 (0.56–2.35)	0.70
*X* ^2^						
Genotype	1.098		1.071		**0.021**	
Allele	0.181		0.193		**0.006**	
Male^‡^	Adjusted OR^‡^		Adjusted OR^‡^		Adjusted OR^‡^	
CC	Ref		Ref		Ref	
AC	1.10 (0.62–1.94)	0.72	1.68 (0.85–3.32)	0.13	0.96 (0.45–2.04)	0.91
AA	1.76 (0.67–4.58)	0.24	0.55 (0.18–1.62)	0.28	0.71 (0.19–2.65)	0.61
Female^‡^						
CC	Ref		Ref		Ref	
AC	0.71 (0.47–1.08)	0.11	0.95 (0.55–1.61)	0.85	0.71 (0.41–1.22)	0.22
AA	0.56 (0.27–1.16)	0.12	1.43 (0.61–3.33)	0.40	1.33 (0.57–3.10)	0.50
Never smoking	Adjusted OR^¶^		Adjusted OR^¶^		Adjusted OR^¶^	
CC	Ref		Ref		Ref	
AC	0.68 (0.46–1.01)	0.61	1.56 (0.91–2.68)	0.10	1.00 (0.58–1.69)	0.99
AA	0.96 (0.49–1.86)	0.90	1.38 (0.62–3.07)	0.42	1.70 (0.73–3.94)	0.21
Ever smoking^†¶^						
CC	Ref		Ref		Ref	
AC	1.34 (0.70–2.57)	0.36	0.80 (0.41–1.57)	0.53	0.60 (0.26–1.37)	0.23
AA	0.73 (0.26–2.03)	0.55	0.48 (0.14–1.61)	0.24	0.58 (0.15–2.25)	0.43

**(f):** 

*APOE*	Adj OR (95% CI)	*p* value	Adj OR (95% CI)	*p* value	Adj OR (95% CI)	*p* value
*rs429358*						
All patients^†^	Adjusted OR^†^		Adjusted OR^†^		Adjusted OR^†^	
TT	Ref		Ref		Ref	
CT	1.06 (0.73–1.54)	0.73	0.84 (0.52–1.36)	0.48	0.84 (0.45–1.12)	0.15
CC	1.79 (0.63–5.05)	0.27	0.43 (0.11–1.64)	0.21	0.64 (0.15–2.7)	0.54
*X* ^2^						
Genotype	1.032		NA		NA	
Allele	0.611		0.399		1.649	
Male^‡^	Adjusted OR^‡^		Adjusted OR^‡^		Adjusted OR^‡^	
TT	Ref		Ref		Ref	
CT	1.22 (0.64–2.33)	0.54	0.73 (0.34–1.53)	0.41	0.86 (0.36–2.05)	0.73
CC	4.76 (0.56–40.48)	0.15	1	—	1	—
Female^‡^						
TT	Ref		Ref		Ref	
CT	0.98 (0.62–1.56)	0.96	0.91 (0.48–1.72)	0.79	0.69 (0.36–1.31)	0.26
CC	1.06 (0.28–3.95)	0.92	0.78 (0.18–3.36)	0.74	2.28 (0.31–16.53)	0.41
Never smoking^¶^	Adjusted OR^¶^		Adjusted OR^¶^		Adjusted OR^¶^	
TT	Ref		Ref		Ref	
CT	1.15 (0.74–1.79)	0.52	0.86 (0.47–1.60)	0.65	0.87 (0.47–1.61)	0.67
CC	0.93 (0.28–5.36)	0.77	0.60 (0.11–3.31)	0.56	5.04 (0.39–63.54)	0.21
Ever smoking^†¶^						
TT	Ref		Ref		Ref	
CT	0.88 (0.43–1.77)	0.72	0.76 (0.34–1.66)	0.49	0.40 (0.14–1.09)	0.07
TT	1	—	1	—	8.56 (0.42–174.7)	0.16
*rs7412*						
All patients						
CC	Ref		Ref			
CT	0.91 (0.55–1.50)	0.72	1.15 (0.63–2.08)	0.64	1.30 (0.69–2.46)	0.41
TT	1	—	1	—	3.41 (0.50–23.19)	0.20
*X* ^2^						
Genotype	NA		NA		NA	
Allele	1.831		0.4281		3.407	
Male^‡^	Adjusted OR^‡^		Adjusted OR^‡^		Adjusted OR^‡^	
CC	Ref		Ref		Ref	
CT	1.13 (0.45–2.82)	0.78	1.87 (0.71–4.92	0.20	2.92 (0.88–9.63)	0.07
TT	1	—	1	—	1.29 (0.01–142.0)	0.91
Female^‡^						
CC	Ref		Ref		Ref	
CT	0.83 (0.45–1.52)	0.56	0.84 (0.39–1.84)	0.68	0.92 (0.41–2.03)	0.83
TT	1	—	1	—	4.03 (0.46–34.93)	0.20
Never smoking^¶^	Adjusted OR^¶^		Adjusted OR^¶^		Adjusted OR^¶^	
CC	Ref		Ref		Ref	
CT	0.97 (0.53–1.76)	0.93	1.40 (0.69–2.83)	0.34	1.64 (0.79–3.40)	0.17
TT	1	—	1	—	2.35 (0.16–33.59)	0.52
Ever smoking^†¶^						
CC	Ref		Ref		Ref	
CT	0.77 (0.30–1.95)	0.58	0.71 (0.23–2.17)	0.55	0.83 (.020–3.33)	0.79
TT	1	—	1	—	8.56 (0.42–174.7)	0.16

**(g):** 

*HNF4A*	Adj OR (95% CI)	*p* value	Adj OR (95% CI)	*p* value	Adj OR (95% CI)	*p* value
*rs1800961*						
All patients^†^	Adjusted OR^†^		Adjusted OR^†^		Adjusted OR^†^	
CC	Ref		Ref		Ref	
CT	0.90 (0.45–1.82)	0.78	**1.96 (1.01–3.80)**	**0.04**	**3.75 (1.64–8.60)**	**0.002** ^∗^
TT	1	—	1	—	1	—
*X* ^2^						
Genotype	NA		NA		NA	
Allele	0.114		4.592		6	
Male^‡^	Adjusted OR^‡^		Adjusted OR^‡^		Adjusted OR^‡^	
CC	Ref		Ref		Ref	
CT	1.50 (0.46–4.92)	0.49	1.48 (0.52–4.21)	0.45	**6.08 (1.25–29.48)**	**0.02**
TT	—	—	—	—	—	—
Female^‡^						
CC	Ref		Ref		Ref	
CT	0.67 (0.27–1.66)	0.39	2.36 (1.01–5.56)	0.04	**3.23 (1.22–8.58)**	**0.01**
TT	1	—	1	—	1	—
Never smoking^¶^	Adjusted OR^¶^		Adjusted OR^¶^		Adjusted OR^¶^	
CC	Ref		Ref		Ref	
CT	0.97 (0.43–2.19)	0.95	1.84 (0.77–4.35)	0.16	**2.96 (1.16–7.53)**	**0.02**
TT	1	—	—	—	1	—
Ever smoking^†¶^						
CC	Ref		Ref		Ref	
CT	0.72 (0.18–2.83)	0.64	2.14 (0.76–6.01)	0.14	6.23 (0.74–52.09)	0.09
TT	1	—	1	—	1	—

Bold font indicates *p* < 0.05; ^∗^indicates statistical significance after Bonferroni correction (*p* < 0.003). ^†^OR with 95% CI were estimated by multiple logistic regression models after controlling for age, sex, and smoking. ^‡^OR with 95% CI were estimated by multiple logistic regression models after controlling for age and smoking. ^¶^OR with 95% CI were estimated by multiple logistic regression models after controlling for age and sex. ^¥^OR with 95% CI were estimated by multiple logistic regression models after controlling for age, considering only females who never smoked. Abbreviations: OR: odds ratio; CI: confidence interval.

**Table 4:** Regression coefficients and 95% CI for effects of SNPs on periodontal parameters (performed by multiple linear regression analysis).

SNP	Number of remaining teeth		Marginal bleeding (% of sites)		BOPi (% of sites)		PPDi ≥ 5 mm (% of sites)		CALi ≥ 6 mm (% of sites)	
	Adjusted *β*^†^ (95% CI)	*p* value	Adjusted *β*^†^ (95% CI)	*p* value	Adjusted *β*^†^ (95% CI)	*p* value	Adjusted *β*^†^ (95% CI)	*p* value	Adjusted *β*^†^ (95% CI)	*p* value
*LDLR*										
rs5925	1.00		1.00		1.00		1.00		1.00	
CT	-0.01 (-0.83–0.06)	0.79	0.01 (-0.06–0.08)	0.71	-0.38 (-2.10–1.34)	0.67	0.04 (-1.78–1.87)	0.96	-0.38 (-2.10–1.34)	0.67
CC	-0.15 (-0.30–<0.01)	0.05	**-0.17 (-0.33–0.24)**	**0.02**	-1.25 (-4.82–2.33)	0.49	0.01 (-3.78–3.80)	1.00	-1.25 (-4.82–2.33)	0.49
rs688										
CC	1.00		1.00		1.00		1.00		1.00	
CT	-0.01 (-0.08–0.04)	0.62	-<0.01 (-0.06–0.06)	0.92	0.01 (-0.03–0.06)	0.56	<0.01 (-0.06–0.06)	0.97	-0.01 (-0.07–0.05)	0.74
TT	-0.02 (-0.11–0.06)	0.60	<0.01 (-0.08–0.09)	0.86	0.01(-0.05–0.08)	0.59	<0.01 (-0.08–0.08)	0.96	-0.02 (-0.11–0.05)	0.50
*APOB*										
rs676210										
GG	1.00		1.00		1.00		1.00		1.00	
AG	0.04 (-0.01–0.11)	0.14	0.05 (-<0.01–0.12)	0.08	**0.05 (**<0.01**–0.10)**	**0.02**	0.05 (-<0.01–0.12)	0.06	**0.07 (0.01–0.14)**	**0.01**
AA	0.01 (-0.11–0.14)	0.82	0.08 (-0.04–0.21)	0.21	0.06 (-0.03–0.17)	0.18	0.07 (-0.05–0.20)	0.24	0.07 (-0.05–0.19)	0.27
rs1042031										
CC	1.00		1.00		1.00		1.00		1.00	
CT	**-0.08 (-0.14–0.01)**	**0.017**	-.05 (-0.11–0.01)	0.13	**-0.05 (-0.10–**<0.01**)**	**0.03**	**-0.08 (-0.14–0.02)**	**0.008**	**-0.07 (-0.13–0.01)**	**0.01**
TT	-0.03 (-0.21–0.15)	0.73	-0.08 (-0.26–0.09)	0.36	-0.13 (-0.28–<0.01)	0.05	-0.11 (-0.28–0.06)	0.20	-0.10 (-.27–0.07)	0.24
rs693										
GG	1.00		1.00		1.00		1.00		1.00	
AG	<0.01 (-0.06–0.06)	0.93	<0.01 (-0.05–0.07)	0.87	-<0.01 (-0.05–0.04)	0.99	<0.01 (-0.05–0.07)	0.76	0.01 (-0.05–0.07)	0.74
AA	-<0.01 (-0.09–0.08)	0.96	-0.02 (-0.11–0.06)	0.52	-0.04 (-0.10–0.02)	0.23	-0.03 (-0.11–0.05)	0.43	-0.06 (-0.15–0.02)	0.13
*ABCC8*										
rs6544718										
CC	1.00		1.00		1.00		1.00		1.00	
CT	-0.02 (-0.09–0.04)	0.45	-0.03 (-0.10–0.03)	0.31	0.01 (-0.03–0.06)	0.61	0.01 (-0.05–0.07)	0.69	0.01 (-0.05–0.07)	0.76
TT	0.10 (-0.09–0.30)	0.29	0.05 (-0.14–0.24)	0.58	0.09 (-0.05–0.24)	0.19	0.06 (-0.12–0.24)	0.52	0.05 (-0.12–0.24)	0.53
rs6544713										
CC	1.00		1.00		1.00		1.00		1.00	
CT	0.05 (-0.01–0.11)	0.10	<0.01 (-0.06–0.06)	0.99	<0.01 (-0.04–0.05)	0.74	-<0.01 (-0.06–0.05)	0.83	-<0.01 (-0.06–0.05)	0.77
TT	0.07 (-0.03–0.18)	0.19	0.03 (-0.07–0.14)	0.56	-<0.01 (-0.09–0.07)	0.82	-0.01 (-0.11–0.09)	0.81	-0.01 (-0.11–0.09)	0.81
*LPL*										
rs285										
TT	1.00		1.00		1.00		1.00		1.00	
CT	-0.02 (-0.09–0.04)	0.50	-0.02 (-0.09–0.04)	0.49	-<0.01 (-0.06–0.04)	0.79	-0.05 (-0.11–0.01)	0.11	-0.04 (-0.10–0.02)	0.22
CC	-0.03 (-0.11–0.05)	0.46	-0.02 (-0.10–0.05)	0.58	<0.01 (-0.06–0.06)	1.00	-0.01 (-0.08–0.06)	0.75	-0.01 (-0.09–0.06)	0.71
rs3735964										
CC	1.00		1.00		1.00		1.00		1.00	
AC	-0.03 (-0.11–0.04)	0.41	-0.01 (-0.08–0.06)	0.77	-0.04 (-0.10–0.01)	0.15	-0.04(-0.11–0.03)	0.26	-0.03 (-0.10–0.04)	0.42
AA	-0.11 (-0.39–0.15)	0.39	-0.08 (-0.35–0.18)	0.54	-0.08 (-0.29–0.11)	0.40	-0.07 (-0.32–0.18)	0.57	-0.07 (-0.33–0.17)	0.54
rs13702										
TT	1.00		1.00		1.00		1.00		1.00	
CT	-0.06 (-0.12–<0.01)	0.06	0.02 (-0.09–0.03)	0.40	-0.01 (-0.06–0.03)	0.62	-0.02 (-0.08–0.04)	0.51	-0.03 (-0.09–0.02)	0.29
CC	-0.02 (-0.12–0.07)	0.62	-0.03 (-0.13–0.06)	0.49	0.02 (-0.10–0.05)	0.53	-0.01 (-0.11–0.08)	0.75	-0.03 (-0.13–0.05)	0.44
*HNF1A*										
rs2650000										
CC	1.00		1.00		1.00		1.00		1.00	
AC	-0.02 (-0.09–0.03)	0.36	-0.04 (-0.11–0.01)	0.13	-0.04 (-0.09–<0.01)	0.06	-0.05 (-0.11–<0.01)	0.09	-0.04 (-0.10–0.01)	0.13
AA	0.02 (-0.08–0.12)	0.69	<0.01 (-0.10–0.10)	0.97	<0.01 (-0.07–0.08)	0.93	-0.01 (-0.10–0.08)	0.82	0.01 (-0.08–0.11)	0.76
*APOE*										
rs429358										
TT	1.00		1.00		1.00		1.00		1.00	
CT	-<0.01 (-0.07–0.06)	0.89	-<0.01 (-0.07–0.06)	0.89	0.01 (-0.03–0.07)	0.55	-<0.01 (-0.06–0.06)	0.96	<0.01 (-0.06–0.07)	0.91
CC	0.07 (-0.11–0.27)	0.42	0.03 (-0.15–0.23)	0.69	0.02 (-0.11–0.17)	0.71	0.10 (-0.07–0.28)	0.26	0.15 (-0.03–0.33)	0.10
rs7412										
CC	1.00		1.00		1.00		1.00		1.00	
CT	-0.03 (-0.13–0.05)	0.41	<0.01 (-0.08–0.10)	0.85	0.02 (-0.04–0.09)	0.45	0.03 (-0.05–0.11)	0.49	0.02 (-0.06–0.11)	0.63
TT	-0.20 (-0.57–0.16)	0.27	-0.25 (-0.61–0.11)	0.17	-0.08 (-0.36–0.19)	0.56	-0.03 (-0.37–0.30)	0.84	-0.11 (-0.46–0.23)	0.51
*HNF4A*										
rs1800961										
CC	1.00		1.00		1.00		1.00		1.00	
CT	0.04 (-0.07–0.16)	0.46	0.04 (-0.07–0.16)	0.48	-0.01 (-0.10–0.08)	0.80	-<0.01 (-0.1–0.11)	0.94	0.02 (-0.08–0.14)	0.64
TT	-0.62 (-1.51–0.27)	0.17	-0.51 (-1.39–0.36)	0.25	-0.38 (-1.06–0.29)	0.26	-0.46 (-1.30–0.36)	0.27	0.47 (-1.32–0.36)	0.26

Bold font indicates *p* < 0.05. ^∗^Indicates statistical significance after Bonferroni's correction (*p* < 0.003). ^†^The regression coefficients with 95% CI were estimated by multiple linear regression models after controlling for age, sex, and smoking. Abbreviations: PD: periodontal disease; *β*: regression coefficient; CI: confidence interval; PPDi: interproximal probing pocket depth; CALi: interproximal clinical attachment level.

**Table 5:** Regression coefficients and 95% CI for effects of SNPs on glycemic, lipid, and obesity parameters (performed by multiple linear regression analysis).

Gene	Fasting glucose		Insulin		HbA1c		Triglycerides		Total cholesterol		HDL		LDL		BMI (kg/m^2^)		Waist-to-hip ratio (cm)	
SNP	Adjusted *β*^†^ (95% CI)	*p* value	Adjusted *β*^†^ (95% CI)	*p* value	Adjusted *β*^†^ (95% CI)	*p* value	Adjusted *β*^†^ (95% CI)	*p* value	Adjusted *β*^†^ (95% CI)	*p* value	Adjusted *β*^†^ (95% CI)	*p* value	Adjusted *β*^†^ (95% CI)	*p* value	Adjusted *β*^†^ (95% CI)	*p* value	Adjusted *β*^†^ (95% CI)	*p* value
*LDLR*																		
rs5925																		
TT	Ref		Ref		Ref		Ref		Ref		Ref		Ref		Ref		Ref	
CT	-0.04 (-0.15–0.06)	0.38	-0.01 (-0.14–0.12)	0.86	-0.02 (-0.12–0.08)	0.66	-0.02 (-0.13–0.08)	0.60	-0.01 (-0.12–0.09)	0.84	-0.004 (-0.11–0.10)	0.93	-0.004 (-0.12–0.11)	0.94	-0.08 (-0.18–0.02)	0.13	**-0.13 (-0.26–0.001)**	**0.05**
CC	0.006 (-0.13–0.14)	0.92	0.02 (-0.15–0.20)	0.76	0.03 (-0.10–0.17)	0.64	-0.01 (-0.16–0.12)	0.80	0.02 (-0.11–0.16)	0.73	0.01 (-0.13–0.16)	0.81	0.02 (-0.13–0.17)	0.77	-0.06 (-0.20–0.07)	0.33	-0.06 (-0.23–0.10)	0.43
rs688																		
CC	Ref		Ref		Ref		Ref		Ref		Ref		Ref		Ref		Ref	
CT	-0.02 (-0.12–0.08)	0.67	0.02 (-0.10–0.15)	0.68	0.004 (-0.09–0.10)	0.93	-0.01 (-0.12–0.09)	0.81	0.01 (-0.09–0.12)	0.81	0.01 (-0.09–0.13)	0.75	0.01 (-0.09–0.13)	0.76	-0.05 (-0.16–0.04)	0.27	-0.10 (-0.23–0.03)	0.13
TT	-0.01 (-0.16–0.13)	0.83	0.002 (-0.18–0.19)	0.98	0.006 (-0.13–0.15)	0.93	-0.03 (-0.17–0.11)	0.64	0.006 (-0.13–0.15)	0.93	-0.008 (-0.16–0.14)	0.91	-0.008 (-0.16–0.14)	0.91	-0.09 (-0.23–0.04)	0.19	-0.09 (-0.26–0.08)	0.29
*APOB*																		
rs676210																		
GG	Ref		Ref		Ref		Ref		Ref		Ref		Ref		Ref		Ref	
AG	0.02 (-0.07–0.13)	0.58	-0.002 (-0.13–0.13)	0.97	0.004 (-0.09–0.10)	0.92	0.01 (-0.08–0.12)	0.71	0.02 (-0.08–0.12)	0.69	0.01 (-0.09–0.12)	0.75	0.008 (-0.10–0.12)	0.88	0.02 (-0.07–0.12)	0.68	-0.01 (-0.14–0.11)	0.80
AA	**0.21 (0.01–0.41)**	**0.03**	0.14 (-0.09–0.39)	0.23	0.17 (-0.01–0.37)	0.07	0.13 (-0.07–0.34)	0.20	0.17 (-0.03–0.38)	0.10	0.17 (-0.03–0.39)	0.10	0.17 (-0.05–0.40)	0.13	0.20 (-0.01–0.43)	0.07	0.24 (-0.04–0.53)	0.09
rs1042031																		
CC	Ref		Ref		Ref		Ref		Ref		Ref		Ref		Ref		Ref	
CT	**-0.14 (-0.26–-0.03)**	**0.008**	**-0.14 (-0.28–-0.01)**	**0.03**	**-0.14 (-0.25–-0.03)**	**0.01**	**-0.13 (-0.25–-0.02)**	**0.01**	**-0.12 (-0.23–-0.01)**	**0.03**	**-0.11 (-0.23–-0.001)**	**0.04**	**-0.12 (-0.24–-0.001)**	**0.04**	**-0.14 (-0.25–-0.03)**	**0.01**	**-0.25 (-0.38–-0.11)**	**<0.01**∗
TT	-0.01 (-0.25–0.21)	0.88	0.19 (-0.26–0.29)	0.89	-0.06 (-0.29–0.16)	0.58	-0.04 (-0.28–0.20)	0.74	0.02 (-0.22–0.27)	0.84	0.006 (-0.24–0.26)	0.95	0.03 (-0.22–0.29)	0.80	-0.02 (-0.27–0.21)	0.82	-0.01 (-0.28–0.25)	0.88
rs693																		
GG	Ref		Ref		Ref		Ref		Ref		Ref		Ref					
AG	0.003 (-0.09–0.10)	0.94	-0.02 (-0.15–0.09)	0.66	<0.01 (-0.09–0.10)	0.98	-0.01 (-0.12–0.08)	0.72	-0.02 (-0.13–0.07)	0.60	-0.03 (-0.14–0.07)	0.53	-0.02 (-0.13–0.08)	0.68	-0.04 (-0.14–0.05)	0.41	-0.06 (-0.18–0.06)	0.34
AA	0.003 (-0.14–0.15)	0.96	0.005 (-0.18–0.19)	0.95	0.02 (-0.12–0.16)	0.77	-0.004 (-0.12–0.08)	0.94	-0.02 (-0.17–0.12)	0.71	-0.02 (-0.18–0.13)	0.76	-0.01 (-0.17–0.14)	0.86	0.07 (-0.07–0.22)	0.31	0.10 (-0.07–0.29)	0.24
*ABCC8*																		
rs6544718																		
CC	Ref		Ref		Ref		Ref		Ref		Ref		Ref		Ref		Ref	
CT	0.01 (-0.09–0.12)	0.74	-0.04 (-0.17–0.09)	0.56	-0.001 (-0.10–0.10)	0.98	-0.03 (-0.13–0.07)	0.57	-0.03 (-0.14–0.07)	0.55	-0.02 (-0.13–0.08)	0.66	-0.01 (-0.12–0.10)	0.83	-0.03 (-0.14–0.06)	0.48	-0.04 (-0.18–0.09)	0.52
TT	0.01 (-0.33–0.36)	0.92	-0.03 (-0.53–0.46)	0.89	0.04 (-0.29–0.37)	0.81	-0.01 (-0.37–0.34)	0.93	0.02 (-0.34–0.38)	0.90	0.01 (-0.36–0.38)	0.95	-0.01 (-0.43–0.40)	0.94	0.005 (-0.33–0.35)	0.97	-0.10 (-0.59–0.38)	0.68
rs6544713																		
CC	Ref		Ref		Ref		Ref		Ref		Ref		Ref		Ref		Ref	
CT	0.03 (-0.07–0.13)	0.53	0.006 (-0.12–0.13)	0.91	0.01 (-0.08–0.11)	0.71	0.02 (-0.08–0.12)	0.69	0.01 (-0.08–0.12)	0.77	0.03 (-0.06–0.14)	0.47	0.05 (-0.05–0.16)	0.35	0.06 (-0.03–0.15)	0.22	0.04 (-0.08–0.16)	0.51
TT	-0.002 (-0.16–0.15)	0.97	-0.01 (-0.21–0.18)	0.87	0.01 (-0.13–0.17)	0.82	-0.05 (-0.22–0.10)	0.48	-0.04 (-0.20–0.12)	0.61	-0.02 (-0.19–0.15)	0.81	-0.02 (-0.19–0.15)	0.80	0.007 (-0.16–0.18)	0.93	-0.01 (-0.24–0.21)	0.90
*LPL*																		
rs285																		
TT	Ref		Ref		Ref		Ref		Ref		Ref		Ref		Ref		Ref	
CT	-0.009 (-0.12–0.10)	0.86	-0.04 (-0.18–0.10)	0.57	0.001 (-0.10–0.11)	0.97	-0.007 (-0.12–0.10)	0.89	0.004 (-0.11–0.12)	0.93	-0.002 (-0.12–0.11)	0.96	-0.007 (-0.13–0.11)	0.90	-0.05 (-0.17–0.05)	0.30	-0.10 (-0.24–0.02)	0.12
CC	-0.04 (-0.16–0.07)	0.46	-0.07 (-0.22–0.08)	0.36	-0.05 (-0.17–0.06)	0.40	-0.05 (0.18–0.06)	0.37	-0.05 (-0.17–0.07)	0.43	-0.05 (-0.18–0.08)	0.45	-0.04 (-0.18–0.08)	0.50	**-0.12 (-0.25–-0.004)**	**0.04**	**-0.16 (-0.32–-0.01)**	**0.03**
rs3735964																		
CC	Ref		Ref		Ref		Ref		Ref		Ref		Ref		Ref		Ref	
AC	-0.01 (-0.15–0.12)	0.84	0.005 (-0.16–0.17)	0.95	-0.01 (-0.14–0.11)	0.82	-0.007 (-0.14–.13)	0.91	-0.01 (-0.14–0.12)	0.87	-0.03 (-0.18–0.11)	0.65	-0.02 (-0.17–0.12)	0.77	0.06 (-0.06–0.19)	0.31	0.09 (-0.06–0.26)	0.25
AA	-0.29 (-0.67–0.09)	0.14	-0.17 (-0.79–0.44)	0.58	-0.28 (-0.66–0.09)	0.13	-0.25 (-0.65–0.15)	0.21	-0.29 (-0.70–0.11)	0.15	-0.27 (-0.69–0.14)	0.19	-0.27 (-0.69–0.14)	0.19	1		1	
rs13702																		
TT	Ref		Ref		Ref		Ref		Ref		Ref		Ref		Ref		Ref	
CT	-0.07 (-0.17–0.02)	0.15	-0.10 (-0.23–0.02)	0.12	-0.07 (-0.17–0.02)	0.14	-0.02 (-0.13–0.07)	0.63	-0.03 (-0.14–0.07)	0.54	-0.03 (-0.15–0.07)	0.49	-0.04 (-0.16–0.06)	0.42	-0.01 (-0.12–0.08)	0.70	-0.02 (-0.16–0.10)	0.65
CC	-0.009 (-0.15–0.13)	0.89	-0.02 (-0.21–0.15)	0.77	-0.04 (-0.18–0.10)	0.57	-0.01 (-0.16–0.12)	0.79	-0.02 (-0.17–0.12)	0.72	-0.02 (-0.18–0.12)	0.70	-0.02 (-0.18–0.13)	0.75	0.02 (-0.12–0.18)	0.71	0.08 (-0.10–0.26)	0.38
*HNF1A*																		
rs2650000																		
CC	Ref		Ref		Ref		Ref		Ref		Ref		Ref		Ref		Ref	
AC	-0.06 (-.16–0.03)	0.18	-0.08 (-0.20–0.04)	0.20	-0.06 (-0.16–0.02)	0.16	-0.07 (-0.17–0.02)	0.12	**-0.10 (-0.20–-0.001)**	**0.04**	-0.10 (-0.20–0.004)	0.06	-0.10 (-0.21–0.0007)	0.05	-0.007 (-0.10–0.09)	0.88	-0.02 (-0.14–0.10)	0.73
AA	-0.01 (-0.17–0.14)	0.87	0.01 (-0.18–0.21)	0.86	0.01 (-0.13–0.17)	0.82	0.01 (-0.14–0.17)	0.83	<0.01 (-0.16–0.16)	0.99	-0.008 (-0.18–0.16)	0.91	0.004 (-0.16–0.17)	0.95	<0.01 (-0.15–0.15)	0.99	0.03 (-0.15–0.22)	0.69
*APOE*																		
rs429358																		
TT	Ref		Ref		Ref		Ref		Ref		Ref		Ref		Ref		Ref	
CT	0.05 (-0.05–0.17)	0.31	0.03 (-0.10–0.17)	0.62	0.04 (-0.06–0.15)	0.43	0.06 (-0.04–0.18)	0.23	0.04 (-0.07–0.16)	0.46	0.07 (-0.04–0.19)	0.23	0.06 (-0.05–0.18)	0.29	0.04 (-0.06–0.15)	0.42	0.05 (-0.09–0.19)	0.48
CC	0.32 (-0.11–0.77)	0.14	0.28 (-0.32–0.89)	0.36	0.34 (-0.08–0.77)	0.11	0.27 (-0.18–0.72)	0.23	0.18 (-0.27–0.65)	0.42	0.24 (-0.22–0.71)	0.30	0.23 (-0.24–0.70)	0.34	0.23 (-0.20–0.67)	0.29	0.28 (-0.31–0.88)	0.34
rs7412																		
CC	Ref		Ref		Ref		Ref		Ref		Ref		Ref		Ref		Ref	
CT	0.01 (-0.14–0.16)	0.88	0.05 (-0.12–0.23)	0.55	-0.01 (-0.16–0.13)	0.85	0.01 (-0.13–0.16)	0.83	0.02 (-0.13–0.18)	0.73	0.01 (-0.14–0.17)	0.82	0.02 (-0.13–0.18)	0.76	-0.001 (-0.14–0.13)	0.98	0.01 (-0.16–0.19)	0.88
TT	-0.08 (-0.47–0.29)	0.64	-0.17 (-0.78–0.43)	0.56	-0.05 (-0.43–0.32)	0.77	-0.12 (-0.58–0.32)	0.58	-0.10 (-0.56–0.36)	0.66	-0.09 (-0.57–0.37)	0.68	-0.09 (-0.57–0.39)	0.71	0.11 (-0.42–0.65)	0.66	0.24 (-0.60–1.08)	0.56
*HNF4A*																		
rs1800961																		
CC	Ref		Ref		Ref		Ref		Ref		Ref		Ref		Ref		Ref	
CT	-0.05 (-0.21–0.10)	0.48	-0.11 (-0.31–0.09)	0.28	-0.05 (-0.21–0.10)	0.52	-0.01 (-0.17–0.14)	0.84	-0.009 (-0.17–0.15)	0.91	-0.02 (-0.19–0.14)	0.80	-0.01 (-0.18–0.15)	0.84	-0.05 (-0.21–0.09)	0.45	-0.07 (-0.26–0.12)	0.46
TT	1		1		1		1		1		1		1		1		1	

Bold font indicates *p* < 0.05. ^∗^Indicates statistical significance after Bonferroni's correction (*p* < 0.003). ^†^The regression coefficients with 95% CI were estimated by multiple linear regression models after controlling for age, sex, and smoking. Abbreviations: PD: periodontal disease; *β*: regression coefficient; CI: confidence interval; PPDi: interproximal probing pocket depth; CALi: interproximal clinical attachment level.

## Data Availability

The genotypes and biochemical data used to support the findings of this study are included within the article and the supplementary information file.

## Supplementary Materials

Supplementary Materials Details of the sample size calculation with the G∗ Power Calculator, version 3.1.9. Supplementary Table 1: allele and genotype frequencies, minor allele frequency (MAF) analysis, and Hardy–Weinberg equilibrium (HWE). Supplementary Figure 1: results in each group of Spearman's correlations between biochemical profiles and periodontal parameters. Supplementary Table 2: Median (minimum–maximum) of glycemic and lipid profiles and physical exams of individuals belonging to the T2DM+P subgroups.
