# Supplementary material for: Polymorphisms in Genes of Lipid Metabolism Are Associated with Type 2 Diabetes Mellitus and Periodontitis, as Comorbidities, and with the Subjects' Periodontal, Glycemic, and Lipid Profiles
Source: J Diabetes Res. 2021 Nov 11;2021:1049307. doi: 10.1155/2021/1049307 (PMC8601849; doi:10.1155/2021/1049307)
Supplement: Supplementary Materials — Details of the sample size calculation with the G∗ Power Calculator, version 3.1.9. Supplementary Table 1: allele and genotype frequencies, minor allele frequency (MAF) analysis, and Hardy–Weinberg equilibrium (HWE). Supplementary Figure 1: results in each group of Spearman's correlations between biochemical profiles and periodontal parameters. Supplementary Table 2: Median (minimum–maximum) of glycemic and lipid profiles and physical exams of individuals belonging to the T2DM+P subgroups. [file 1049307.f1.pdf]

Supplementary Materials

Polymorphisms in genes of lipid metabolism are associated with type 2 Diabetes Mellitus and Periodontitis, as comorbidities, and with the subjects' periodontal, glycemic and lipid profiles

Ingra G. Nicchio,[1,5*] Thamiris Cirelli,[1,5*] Rafael Nepomuceno,[1,5] Marco A. R. Hidalgo,[1,5] Carlos Rossa Jr,[1] Joni A. Cirelli,[1] Silvana R. P. Orrico,[1,2] Silvana P. Barros,[3] Letícia H. Theodoro,[4] and Raquel M. Scarel-Caminaga,[5].

Material and Methods

To calculate the sample size needed for the study to have power to attest the association between the alleles and periodontitis, the G* Power Calculator, version 3.1.9 (Faul et al., 2007) software was used, considering the parameters: logistic regression; two tail, OR: 1.5; Pr (Y = 1 / X = 1) H0 = 0.2; alpha of 0.003 (0.05/ 14 SNPs) and 80% power, R2 = 0. This calculation resulted in a total sample of 572 subjects, being 190 individuals per group. Therefore, the genetic analyses in this study have a power greater than 80% to attest the association of the allele(s) with the diseased phenotype, since each studied group comprises more than 190 subjects.

Results

The Supplementary Table 1 showed the allele and genotype frequencies of each SNP that was efficiently genotyped in each group, according to the call rate data in the Table 1. Excepting for the rs1800961 SNP in Healthy and Periodontitis groups, all other minor allele frequency (MAF) were ≥ 5%. The Hardy–Weinberg Equilibrium (HWE) was calculated for each group, and it is possible to observe that the SNP rs285 in the T2DM+P group was not in the HWE, suggesting a potential association of this genotype with the diseases.

Supplementary Table 1. Alleles and genotypes frequencies, Minor Allele Frequency (MAF) analysis and Hardy–Weinberg Equilibrium (HWE)

	**HEALTHY n=343**	**PERIODONTITIS n= 345**	**T2DM+P n= 205**
LDLR			
**rs5925**	n (%)	n (%)	n (%)
Alleles T/C	406 (59.9) /272 (40.1)	413 (60.2) /273 (39.8)	216 (59.7)/146 (40.3)
MAF	0.40	0.40	0.40
Genotypes	n (%)	n (%)	n (%)
TT	124 (36.6)	128 (37.3)	68 (37.6)

CT	158 (40.6)	157 (45.8)	80 (44.2)
CC	57 (16.8)	58 (19.9)	33 (18.2)
HWE	0.57	0.43	0.36
**rs688**	n (%)	n (%)	n (%)
Alleles C/T	255 (37.5)/ 425 (62.5)	429 (63.3)/ 249 (36.7)	230 (63.5)/ 132 (36.5)
MAF	0.37	0.37	0.36
Genotypes	n (%)	n (%)	n (%)
CC	137 (40.3)	141 (41.6)	78 (43.1)
CT	151 (44.4)	147 (43.4)	74 (40.9)
TT	52 (15.3)	51 (15.0)	29 (16.0)
HWE	0.35	0.24	0.16
APOB			
**rs676210**	n (%)	n (%)	n (%)
Alleles G/A	549 (81.0)/ 129 (19.0)	533 (77.9)/ 151 (22.1)	302 (79.9)/ 76 (20.1)
MAF	0.19	0.22	0.20
Genotypes			
GG	228 (67.3)	213 (62.3)	120 (63.5)
GA	93 (27.4)	107 (31.3)	62 (32.8)
AA	18 (5.3)	22 (6.4)	7 (3.7)
HWE	0.05	0.11	1.00
**rs1042031**	n (%)	n (%)	n (%)
Alleles C/T	544 (80.2)/ 134 (19.8)	575 (83.6)/ 113 (16.4)	308 (84.6)/ 56 (15.4)
MAF	0.20	0.16	0.15
Genotypes			
CC	214 (63.1)	238 (69.2)	134 (73.6)
CT	116 (34.2)	99 (28.8)	40 (22.0)
TT	9 (2.7)	7 (2.0)	8 (4.4)
HWE	0.17	0.43	0.05
**rs693**	n (%)	n (%)	n (%)
Alleles G/A	421 (61.4)/ 265 (38.6)	421 (61.9)/ 259 (38.1)	242 (62.7)/ 144 (37.3)
MAF	0.39	0.38	0.37
Genotypes			
GG	136 (39.6)	133 (39.1)	76 (39.4)
GA	149 (43.4)	155 (45.6)	90 (46.6)
AA	58 (17.0)	52 (15.3)	27 (14.0)
HWE	0.13	0.56	1.00
ABCC8			
**rs6544718**	n (%)	n (%)	n (%)
Alleles C/T	574 (83.80) /110 (16.20)	574 (83.90) / 112 (16.10)	332 (82.20) / 72 (17.80)
MAF	0.15	0.16	0.17
Genotypes	n (%)	n (%)	n (%)

CC	239 (70.0)	242 (70.55)	133 (65.85)
CT	96 (28.0)	90 (26.25)	66(32.70)
TT	7 (2.0)	11(3.20)	3 (1.45)
HWE	0.55	0.43	0.14
**rs6544713**	n (%)	n (%)	n (%)
Alleles C/T	505 (73.83) /179 (26.17)	496 (76.14) / 192 (23.86)	287 (77.33) / 115 (22.67)
MAF	0.26	0.27	0.28
Genotypes	n (%)	n (%)	n (%)
TT	190 (55.55)	182 (56.29)	104 (51.72)
CT	125 (36.55)	132 (39.71)	79 (38.90)
CC	27 (7.90)	30(4.00)	18 (9.38)
HWE	0.32	0.42	0.60
LPL			
**rs285**	n (%)	n (%)	n (%)
Alleles T/C	351 (51.46) /331 (48.54)	372 (56.86) / 306 (45.14)	201 (52.75) / 185 (47.25)
MAF	0.48	0.45	0.47
Genotypes	n (%)	n (%)	n (%)
TT	94 (55.55)	108 (56.29)	60 (51.72)
CT	163(36.55)	156 (39.71)	81 (38.90)
CC	84 (7.90)	75 (4.00)	52 (9.38)
HWE	0.44	0.18	0.03
**rs3735964**	n (%)	n (%)	n (%)
Alleles C/A	600 (88.23) /80 (11.77)	613 (90.14) / 67 (9.86)	337 (90.10) / 37 (9.10)
MAF	0.11	0.09	0.09
Genotypes	n (%)	n (%)	n (%)
CC	266 (78,23)	278 (81.77)	150 (80.20)
AC	68(20.00)	57 (16.76)	37 (19.80)
AA	6 (1.77)	5 (1.47)	0 (0.0)
HWE	0.43	0.35	0.22
**rs13702**	n (%)	n (%)	n (%)
Alleles T/C	393 (63.80) /233 (36.20)	418 (65.10) / 224 (35.90)	232 (65.90) / 120 (34.10)
MAF	0.37	0.34	0.34
Genotypes	n (%)	n (%)	n (%)
TT	119(38.02)	134 (41.75)	79 (44.85)
CT	155(49.52)	150 (46.72)	74 (42.04)
CC	39 (12.46)	37 (11.53)	23 (13.10)
HWE	0.33	0.71	0.40
HNF1A			
**rs2650000**	n (%)	n (%)	n (%)
Alleles C/A	457 (67.01) /225 (32.09)	467 (68.70) / 217 (31.30)	270 (67.83) / 128 (32.17)
MAF	0.32	0.31	0.32

Genotypes	n (%)	n (%)	n (%)
CC	148 (43.40)	160 (47.05)	92 (46.23)
AC	161 (47.20)	147 (43.23)	86 (43.21)
AA	32 (9.40)	35 (9.72)	21 (10.57)
HWE	0.26	0.90	0.87
APOE			
**rs429358**	n (%)	n (%)	n (%)
Alleles T/C	594 (86.30) /92 (13.70)	579 (84.89) / 103 (15.11)	354 (87.62) / 50 (12.38)
MAF	0.13	0.15	0.12
Genotypes	n (%)	n (%)	n (%)
TT	257 (74.92)	249 (73.02)	155(76.73)
CT	80(23.32)	81 (23.75)	44 (21.78)
CC	6 (1.76)	11 (3.23)	3 (1.49)
HWE	1	0.20	1
**rs7412**	n (%)	n (%)	n (%)
Alleles C/T	637 (93.10) /47 (6.90)	655 (94.90) / 35 (5.10)	371 (92.30) / 31 (7.70)
MAF	0.06	0.05	0.07
Genotypes	n (%)	n (%)	n (%)
CC	298 (87.13)	310 (89.9)	173 (86.10)
CT	41(12.00)	35 (10.10)	25 (12.45)
TT	3 (0.87)	0 (0.00)	3 (1.45)
HWE	0.20	1	0.09
HNF4A			
**rs1800961**	n (%)	n (%)	n (%)
Alleles C/T	656 (97.00) /20 (3.0)	664 (97.36) / 18 (2.64)	385 (94.36) / 23 (5.64)
MAF	0.02	0.02	0.05
Genotypes	n (%)	n (%)	n (%)
CC	319 (94.37)	323 (94.72)	181 (88.72)
CT	18 (5.32)	18 (5.28)	23 (11.28)
TT	1 (0.31)	0 (0.00)	0 (0.00)
HWE	0.25	1	1

HWE: Hardy-Weinberg Equilibrium; MAF: Minor Allele Frequencies

In the Table 3 presented in the main text, when the Periodontitis group was compared to the T2DM+P group, theoretically it could infer the association of each SNP with the T2DM, but because we did not have a group of subjects affected only by T2DM, it is not possible to affirm the associations. On the other hand, when it is find a statistically significant association in the (Periodontitis versus T2DM+P) comparison, we are able to affirm that it was found association with T2DM and Periodontitis as comorbidities (T2DM+P). This occurred with

the *HNF4A*-rs180096-CT which showed more than three times increased susceptibility to T2DM+P (OR = 3.75; CI 95%= 1.64 – 8.60; p = 0.002) for all studied population. Stratifying subjects by sex and by smoking habits, the significant association of this gene marker is maintained, but presenting a large CI, which could be influenced by a decreased sample size, which make the genetic association less plausible.

Spearman's correlations among glycemic, lipid and periodontal parameters were made for each group as demonstrated in the Supplementary Figure 1. Positive (directly proportional) or negative (inversely proportional) statistically significant correlations of biochemical profile and periodontal parameters were found for each group and these results were highlighted. For Healthy group we observed that Fasting Glucose was directly proportional to Insulin and HbA1c levels, as well with BMI, while between the HDL Cholesterol and Triglycerides levels the correlation was inversely proportional. Even being low, the Waist-to-Hip ratio was correlated with BMI, visible plaque index (VPI) and marginal bleeding (MB).

Patients belonging to the Periodontitis group showed statistically significant positive correlation between Fasting Glucose and Waist-to-Hip ratio, as well as between LDL and bleeding on probing (BOP). There were expected correlations among periodontal parameters each other. In regard to the T2DM+P, there was the expected correlation between fasting glucose and HbA1c, besides the same level of correlation of the VPI with MB, bleeding on probing (BOP), probing pocket depth (PPD)≥ 5 mm and clinical attachment level (CAL)≥ 6 mm (Supplementary Figure 1).

Supplementary Figure 1. Results in each group of the Spearman's correlations between biochemical profiles and periodontal parameters

HEALTHY group

	FG	Insulin	HBA1C	Triglyc	Total Chol	HDL - Chol	LDL - Chol	W-H ratio	BMI	VPI	MB	BOPi	PPDi≤4	PPDi≥5	CALi≥3	CALi4-5	CALi≥6
Fasting Glucose		0,034	0,027	0,595	0,564	0,296	0,922	0,253	0,044	0,302	0,14	0,291	0,861	0,861	0,697	0,85	0,104
Insulin	0,355		0,255	0,172	0,102	0,72	0,477	0,369	0,04	0,061	0,345	0,667	0,165	0,165	0,983	0,983	
HBA1C	0,3	-0,195		0,554	0,9	0,234	0,945	0,779	0,912	0,52	0,415	0,371	0,053	0,053	0,865	0,647	0,275
Triglycerides	0,092	0,236	-0,102		0,103	0,004	0,724	0,739	0,922	0,258	0,347	0,918	0,285	0,285	0,556	0,556	
Total Cholesterol	0,099	0,281	0,022	0,268		0,952	0	0,627	0,297	0,1	0,386	0,758	0,564	0,564	0,736	0,736	
HDL - Cholesterol	0,179	0,063	0,204	-0,454	-0,01		0,129	0,133	0,897	0,356	0,085	0,704	0,929	0,929	0,431	0,431	
LDL - Cholesterol	-0,017	0,124	-0,012	0,059	0,868	-0,251		0,99	0,172	0,073	0,7	0,674	0,987	0,987	0,774	0,774	
Wais to Hip Ratio	0,196	0,184	0,048	-0,072	-0,105	-0,316	-0,003		0,001	0,008	0,034	0,098	0,66	0,66	0,484	0,945	0,098
BMI	0,347	0,423	0,02	-0,022	0,227	-0,029	0,295	0,497		0,121	0,079	0,07	0,626	0,626	0,023	0,127	0,106
VPI	-0,143	0,311	0,089	0,188	-0,271	-0,154	-0,294	0,407	0,252		0	0,082	0,014	0,014	0,001	0,017	0,035
MB	-0,204	-0,16	-0,113	-0,157	-0,145	-0,283	0,065	0,331	0,284	0,477		0	0,442	0,442	0,874	0,843	0,002
BOPi	-0,146	-0,073	0,124	-0,017	-0,052	-0,064	-0,071	0,262	0,293	0,285	0,35		0,001	0,001	0	0	0,01
PPDi≤4	-0,024	-0,233	0,265	-0,178	-0,097	-0,015	-0,003	0,071	-0,081	-0,133	-0,189	-0,181		0	0	0,027	0,280
PPDi≥5	0,024	0,233	-0,265	0,178	0,097	0,015	0,003	-0,071	0,081	0,133	0,189	0,181	-1.000		0	0,027	0,280
CALi≤3	-0,054	0,004	0,024	-0,099	-0,057	-0,132	0,048	-0,113	-0,362	-0,174	-0,262	-0,457	0,372	-0,372		0	0,095
CALi4-5	-0,026	-0,004	-0,064	0,099	0,057	0,132	-0,048	0,011	0,249	0,129	0,211	0,433	-0,314	0,314	-0,954		0,744
CALi≥6	0,224		0,151					0,262	0,262	0,114	0,169	0,139	-0,224	0,224	-0,283	0,018	

PERIODONTITIS group

	FG	Insulin	HBA1C	Triglyc	Total Chol	HDL - Chol	LDL - Chol	W-H ratio	BMI	VPI	MB	BOPi	PPDi≤4	PPDi≥5	CALi≥3	CALi4-5	CALi≥6
Fasting Glucose		0,039	0,119	0,173	0,541	0,087	0,997	0,004	0,229	0,117	0,051	0,262	0,827	0,827	0,820	0,594	0,829
Insulin	0,289		0,396	0,006	0,287	0,021	0,633	0,135	0,344	0,598	0,746	0,776	0,707	0,707	0,333	0,052	0,602
HBA1C	0,221	0,121		0,729	0,760	0,502	0,991	0,373	0,629	0,523	0,803	0,665	0,412	0,412	0,933	0,969	0,644
Triglycerides	0,194	0,383	-0,050		0,050	0,048	0,782	0,169	0,349	0,645	0,345	0,831	0,512	0,512	0,875	0,656	0,379
Total Cholesterol	0,088	0,152	-0,044	0,270		0,297	0,003	0,663	0,865	0,957	0,041	0,016	0,647	0,647	0,056	0,058	0,177
HDL - Cholesterol	-0,242	-0,323	0,096	-0,273	0,146		0,144	0,118	0,188	0,499	0,567	0,919	0,829	0,829	0,840	0,955	0,591
LDL - Cholesterol	0,001	0,069	-0,002	0,039	0,708	-0,203		0,964	0,165	0,800	0,128	0,016	0,612	0,612	0,035	0,031	0,159
Wais to Hip Ratio	0,481	0,261	0,158	0,242	0,078	-0,274	0,008		0,056	0,411	0,840	0,278	0,019	0,019	0,504	0,758	0,577
BMI	0,212	0,168	0,086	0,166	-0,030	-0,231	-0,243	0,326		0,607	0,201	0,259	0,420	0,420	0,114	0,243	0,096
VPI	-0,222	-0,076	-0,092	0,065	0,008	0,095	0,036	-0,139	0,085		0,000	1,580	0,015	0,015	0,390	0,001	0,002
MB	-0,275	0,046	-0,036	0,132	0,282	0,080	0,212	0,034	0,209	0,426		0,147	0,479	0,479	0,239	0,303	0,303
BOPi	-0,160	-0,041	-0,062	0,030	0,329	0,014	0,331	-0,183	-0,185	0,278	0,203		0,000	0,000	0,000	0,000	0,000
PPDi≤4	-0,031	-0,054	-0,117	0,092	0,064	-0,030	0,071	0,384	-0,133	-0,131	-0,038	-0,601		0,000	0,000	0,000	0,000
PPDi≥5	0,031	0,054	0,117	-0,092	-0,064	0,030	-0,071	-0,384	0,133	0,131	0,038	0,601	-1,000		0,000	0,000	0,000
CALi≤3	0,033	0,138	0,012	0,022	-0,264	0,028	-0,291	0,113	0,257	-0,190	-0,064	-0,866	0,641	-0,641		0,000	0,000
CALi4-5	-0,076	-0,273	-0,006	-0,063	0,262	0,008	0,296	-0,052	-0,191	0,171	0,056	0,777	-0,476	0,476	-0,883		0,000
CALi≥6	0,031	0,075	0,066	0,123	0,188	-0,076	0,196	-0,095	-0,271	0,169	0,056	0,695	-0,629	0,629	-0,838	0,539	

T2DM+P group

	FG	Insulin	HBA1C	Triglyc	Total Chol	HDL - Chol	LDL - Chol	W-H ratio	BMI	VPI	MB	BOPi	PPDi≤4	PPDi≥5	CALi≥3	CALi4-5	CALi≥6
Fasting Glucose		0,869	0,000	0,004	0,097	0,016	0,969	0,002	0,558	0,021	0,093	0,192	0,003	0,003	0,177	0,116	0,049
Insulin	0,017		0,571	0,895	0,293	0,883	0,805	0,352	0,218	0,630	0,545	0,604	0,768	0,768	0,487	0,175	0,695
HBA1C	0,754	0,057		0,216	0,482	0,324	0,893	0,011	0,885	0,320	0,402	0,697	0,037	0,037	0,571	0,095	0,218
Triglycerides	0,241	0,013	0,103		612,108	2,917	0,057	0,052	0,599	0,010	0,064	0,007	0,003	0,003	0,001	0,069	0,004
Total Cholesterol	0,138	0,105	0,058	0,306		0,932	0,000	0,889	0,428	0,018	0,479	0,110	0,002	0,002	0,380	0,593	0,133
HDL - Cholesterol	-0,203	-0,015	-0,084	-0,398	0,007		0,406	0,277	0,028	0,118	0,290	0,011	0,149	0,149	0,001	0,063	0,005
LDL - Cholesterol	-0,003	0,025	0,012	0,156	0,782	-0,069		0,489	0,024	0,335	0,740	0,622	0,030	0,030	0,645	0,868	0,367
Wais to Hip Ratio	0,321	-0,105	0,264	0,199	0,014	-0,462	0,073		0,028	0,030	0,079	0,114	0,029	0,029	0,064	0,051	0,246
BMI	0,069	0,160	-0,017	0,060	0,090	-0,252	0,257	0,251		0,638	0,842	0,870	0,361	0,361	0,831	0,859	0,541
VPI	0,190	-0,049	0,082	0,202	0,186	-0,127	0,081	0,207	-0,051		0,000	0,000	0,001	0,001	0,035	0,002	0,013
MB	0,139	-0,062	0,069	0,146	0,056	-0,086	0,028	0,168	0,022	0,585		0,563	0,141	0,141	0,538	0,029	0,812
BOPi	0,108	-0,053	0,032	0,212	0,126	-0,207	0,041	0,151	-0,018	0,478	0,349		0,000	0,000	0,000	0,000	0,000
PPDi≤4	-0,246	0,030	-0,170	-0,232	-0,247	0,118	-0,180	-0,208	-0,099	-0,418	-0,392	-0,765		0,000	0,000	4,015	0,000
PPDi≥5	0,246	-0,030	0,170	0,232	0,247	-0,118	0,180	0,208	0,099	0,418	0,392	0,765	-1,000		0,000	4,015	0,000
CALi≤3	-0,112	0,071	-0,046	-0,261	-0,069	0,269	-0,039	-0,177	-0,023	-0,407	-0,350	-0,901	0,728	-0,728		0,000	0,000
CALi4-5	-0,130	-0,138	-0,136	0,144	-0,042	-0,151	-0,014	0,187	-0,019	0,221	0,156	0,639	-0,353	0,353	-0,694		0,382
CALi≥6	0,163	-0,040	0,101	0,227	0,118	-0,228	0,075	0,112	0,066	0,417	0,313	0,792	-0,755	0,755	-0,880	0,381	

Values above light gray meant p values. The green colors meant that there was a positive significant correlation, and the red color meant negative significant correlation. Upper diagonal = p values. Lower diagonal values = ρ=Spearman's correlations coefficients. FG = Fasting Glucose; Triglyc = Triglycerides; Total Chol = Total Cholesterol; HDL Chol = HDL Cholesterol; W-H ratio = Waist to Hip Ratio; BMI = Body mass index; VPI = Visible Plaque index; MB = Marginal Bleeding; BOPi = Bleeding on Probing interproximal; PPDi = Probing Pocket Depth; CALi = Clinical Attachment Level.

We subdivided T2DM+P by the severity of periodontitis and according to the glycemic metabolic control. The Supplementary Table 2 showed that the majority of patients with severe periodontitis were also HbA1c poorly-controlled T2DM (n=84). For both T2DM+P (moderate or severe), some glycemic and lipid biochemical levels were worst in the poorly-controlled T2DM+P.

Supplementary Table 2. Median (minimum - maximum) of glycemic and lipid profiles and physical exams of individuals belonging to the T2DM+P subgroups

	T2DM+P (moderate periodontitis)			***T2DM+P (severe periodontitis)***			
	Well-Controlled ***** *(n= 36)*	*Poorly-Controlled* ***** *(n= 39)*	*p value******	*Well-Controlled* ****** *(n= 46)*	*Poorly-Controlled* ****** *(n= 84)*	*p value*******	*p value* [ab] *among the 4 groups*
**Fasting Glucose**	111.0 (90.0 – 149.0) [a]	176.0 (84.0 – 400.0) [b]	**0.0001**	118.0 (83.00 - 215.0) [a]	198.0 (87.00 - 467.9) [b]	**0.0001**	**0.0001**
**HbA1c**	6.50 (4.50 - 7.00) [a]	8.50 (7.10 - 13.40) [b]	**0.0001**	6.20 (4.70 - 6.90) [a]	9.35 (7.10 - 14.30) [b]	**0.0001**	**0.0001**
**Insulin**	11.70 (6.03 - 47.30) [a]	15.00 (4.80 - 161.0) [a]	0.2770	13.90 (3.20 - 52.60) [a]	13.50 (5.10 - 101.9) [a]	0.8809	0.7036
**Total-Cholesterol**	155.6 (111.0 - 221.0) [a]	204.0 (104.0 - 275.0) [b]	**0.0021**	216.0 (131.9 - 306.0)[b]	188.0 (118.0 - 304.0) [b]	**0.0283**	**0.0005**
**HDL Cholesterol**	45.50 (25.00 - 69.00) [a]	41.00 (29.00 - 72.00) [a]	0.4373	45.00 (30.00 - 75.00) [a]	42.30 (24.00 - 87.00) [a]	0.3124	0.5568
**LDL Cholesterol**	75.60 (30.80 - 142.4) [a]	121.5 (45.40 - 190.4) [a]	**0.0022**	128.6 (33.26 - 209.6) [b]	102.8 (7.50 - 523.0) [a]	0.1955	**0.0409**
**Triglycerides**	140.0 (47.00 - 283.0) [a]	174.5 (57.00 - 440.0) [a]	0.1207	171.0 (61.00 - 439.0) [a]	155.0 (58.90 - 765.0) [a]	0.7153	0.4249
**BMI (Kg/m2)**	27.28 (22.48 - 44.98) [a]	30.85 (23.42 - 47.27) [a]	**0.0361**	30.85 (21.93 - 47.27) [a]	29.34 (21.64 - 41.37) [a]	0.3240	0.1647
**Waist-to-Hip-Ratio (cm)**	0.91 (0.76 - 1.17) [a]	0.96 (0.87 - 1.15) [a]	0.1272	0.94 (0.84 - 1.06) [a]	0.99 (0.86 - 1.25) [a]	0.0327	0.0843

*P<0.05 between well-controlled and poor-controlled patients with moderate periodontitis (Mann Whitney Test, α=5%);

**P<0.05 between well-controlled and poor-controlled patients with severe periodontitis (Mann Whitney Test, α=5%);

[a,b,c] Different letter means p value <0.05 considering the comparison among the four groups (Kruskal-Wallis, followed by Dunn´s Test, α= 5%)
